# New biomarkers underlying acetic acid tolerance in the probiotic yeast *Saccharomyces cerevisiae* var. *boulardii*

**DOI:** 10.1007/s00253-023-12946-x

**Published:** 2024-01-19

**Authors:** Wiwan Samakkarn, Paul Vandecruys, Maria Remedios Foulquié Moreno, Johan Thevelein, Khanok Ratanakhanokchai, Nitnipa Soontorngun

**Affiliations:** 1 Excellent Research Laboratory for Yeast Innovation, Division of Biochemical Technology, School of Bioresources and Technology, King Mongkut’s University of Technology Thonburi, Bangkok, Thailand; 2 Laboratory of Molecular Cell Biology, Institute of Botany and Microbiology, KU Leuven, Leuven, Heverlee Belgium; 3 Center for Microbiology, VIB, Leuven, Flanders Belgium; 4 NovelYeast Bv, Open Bio-Incubator, Erasmus High School, (Jette), Brussels, Belgium; 5 Pilot Plant Development and Training Institute, King Mongkut’s University of Technology Thonburi, Bangkok, Thailand

**Keywords:** Acetic acid tolerance, Food and alcohol fermentation, Microbial stress response, Omic analysis, Probiotics, *Saccharomyces cerevisiae* var. *boulardii*

## Abstract

**Abstract:**

Evolutionary engineering experiments, in combination with omics technologies, revealed genetic markers underpinning the molecular mechanisms behind acetic acid stress tolerance in the probiotic yeast *Saccharomyces cerevisiae* var. *boulardii*. Here, compared to the ancestral Ent strain, evolved yeast strains could quickly adapt to high acetic acid levels (7 g/L) and displayed a shorter lag phase of growth. Bioinformatic-aided whole-genome sequencing identified genetic changes associated with enhanced strain robustness to acetic acid: a duplicated sequence in the essential endocytotic *PAN1* gene, mutations in a cell wall mannoprotein (*dan4*^Thr192del^), a lipid and fatty acid transcription factor (*oaf1*^Ser57Pro^) and a thiamine biosynthetic enzyme (*thi13*^Thr332Ala^). Induction of *PAN1* and its associated endocytic complex *SLA1* and *END3* genes was observed following acetic acid treatment in the evolved-resistant strain when compared to the ancestral strain. Genome-wide transcriptomic analysis of the evolved Ent acid-resistant strain (Ent ev16) also revealed a dramatic rewiring of gene expression among genes associated with cellular transport, metabolism, oxidative stress response, biosynthesis/organization of the cell wall, and cell membrane. Some evolved strains also displayed better growth at high acetic acid concentrations and exhibited adaptive metabolic profiles with altered levels of secreted ethanol (4.0–6.4% decrease), glycerol (31.4–78.5% increase), and acetic acid (53.0–60.3% increase) when compared to the ancestral strain. Overall, duplication/mutations and transcriptional alterations are key mechanisms driving improved acetic acid tolerance in probiotic strains. We successfully used adaptive evolutionary engineering to rapidly and effectively elucidate the molecular mechanisms behind important industrial traits to obtain robust probiotic yeast strains for myriad biotechnological applications.

**Key points:**

•*Acetic acid adaptation of evolutionary engineered robust probiotic yeast S. boulardii*

•*Enterol ev16 with altered genetic and transcriptomic profiles survives in up to 7 g/L acetic acid*

•*Improved acetic acid tolerance of S. boulardii ev16 with mutated PAN1, DAN4, OAF1, and THI13 genes*

**Graphical Abstract:**

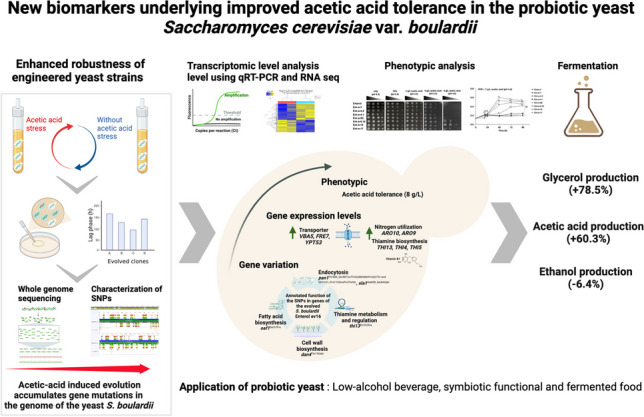

**Supplementary Information:**

The online version contains supplementary material available at 10.1007/s00253-023-12946-x.

## Introduction

Yeast cell factories are versatile platforms that enable the production of highly valuable biochemicals and biofuels due to their robust performance, stress tolerance, safety, and ease of manipulation (Kavšček et al. 2015). *Saccharomyces cerevisiae* var. *boulardii* (*S. boulardii*) belongs to the species *S. cerevisiae* and finds frequent applications in the food and pharmaceutical industries (Khatri et al. 2017; Nandy and Srivastava 2018). Uniquely, *S. boulardii* can tolerate the harsh gastric conditions of the human stomach, showing optimal growth at 37 °C and tolerance to low pH (Hossain et al. 2020). It also produces antimicrobial acids, such as acetic acid (Sen and Mansell 2020). Recently, *S. boulardii* has emerged as a promising microbial cell factory to produce health-beneficial substances, such as the antibacterial peptide leucocin C (Li et al. 2021) or neoagaro-oligosaccharides (Jin et al. 2021). Although the stress response mechanisms in *S. cerevisiae* are well characterized (Giannattasio et al. 2013), strain-to-strain differences exist. Less is known about *S. boulardii*; thus, the investigation of its viability under acid stress may have potential applications in health and biotechnology.

Low-pH environments are harmful to yeast cells due to the dissociated forms of weak acids that could disrupt the cell wall, plasma membrane homeostasis, and proteome functions, including lipid organization and protein conformation (Beales 2004; Guan and Liu 2020). The compromised integrity of the plasma membrane leads to increased passive diffusion of protons, ions, and other small molecules from the environment to the cytosol, inevitably disturbing intracellular homeostasis and cell viability (Liu et al. 2015). Resistance to environmental stress involves a wide range of cellular processes, such as membrane stabilization, glycerol biosynthesis, protein refolding, modification of signaling pathways, regulation of transcription factors associated with cell proliferation, and metabolic activities (Daum et al. 1998; Samakkarn et al. 2021). Genetic engineering and transcriptomic analysis can expand our understanding of the molecular mechanisms underlying the yeast acid stress response and deliver important clues toward improving stress tolerance, bioproduction, and general fermentation performance (Winkler et al. 2013). Acid-tolerant yeasts can be engineered by reprogramming metabolic pathways that support pH homeostasis and trigger stress responses. Several studies have been conducted to understand and improve the response of *S. cerevisiae* to acetic acid. Many proteins associated with transcription control, pH homeostasis, metabolism, and organelle structure and maintenance have been identified; however, the underlying mechanisms of acetic acid tolerance remain unknown (Li and Yuan 2010; Mira et al. 2010). Multiple alleles that affect acetic acid tolerance in this yeast have also been identified (Meijnen et al. 2016; Stojiljković et al. 2022), namely, *HAA1*, *GLO1*, *DOT5*, *CUP2*, *SNF4*, and *VMA7*.

Many stress-responsive transcription factors associated with the reprogramming of gene expression in *S. cerevisiae* have been previously reported (Soontorngun 2017). The transcription factor Haa1 has a pivotal role, as it regulates the acetic acid stress response genes *TPO2* and *TPO3*, which are required to maintain plasma membrane integrity and cell wall structure; the putative membrane protein *YRO2*; and the cell wall-related secretory glycoprotein *YGP1* (Fernandes et al. 2005). Expression of these acid-responsive genes increases with overexpression of *HAA1* (Tanaka et al. 2012). In addition, when *S. cerevisiae* is exposed to acetic acid or ethanol, the zinc cluster transcription factor Znf1 upregulates the expression of the plasma membrane H^+^-ATPase gene, *PMA1*, and the genes linked to the unfolded protein response, the heat shock response, glycerol and carbohydrate metabolism, and the biosynthesis of cellular components (Samakkarn et al. 2021; Songdech et al. 2020; Tangsombatvichit et al. 2015). Responses to nonfermentable carbon sources and high salt concentrations are also governed by the regulator Znf1 (Samakkarn et al. 2021; Songdech et al. 2020; Tangsombatvichit et al. 2015). Furthermore, Znf1 confers tolerance to lignocellulosic inhibitors, furfural and levulinic acids. This tolerance is considered an advantage when using lignocellulosic biomass for biofuel and biochemical production (Songdech et al. 2022). When compared to *S. cerevisiae*, little is known about the regulation of the stress response in *S. boulardii*. In addition, the expression of genes encoding some zinc finger transcription factors, namely, Usv1, Adr1, Gzf3, Hap1, and the polyamine transporter Tpo2, is upregulated in *S. boulardii* under simulated gastrointestinal (GI)-tract conditions (Pais et al. 2021).

Adaptive laboratory engineering is a powerful tool in industrial strain improvement. It enables the creation of superior yeast strains in a controlled laboratory setting, even when the genetic basis underlying the desired trait is not known (Winkler et al. 2013). Laboratory experimental evolution mimics the natural accumulation of adaptive mutations in organisms in response to specific stress conditions (Lee and Kim 2020); it involves several events of mutagenesis and recombination or gene shuffling to optimize microbial performance (Nevoigt 2008; Sauer 2001). Mutant strains and global gene expression changes are commonly evaluated using next-generation sequencing technologies such as whole-genome sequencing (WGS) and RNA sequencing (RNA-Seq) (Yang et al. 2020). The accumulation of genetic polymorphisms in genomes has diverse evolutionary implications (Guillamón and Barrio 2017). A wide range of genetic alterations, including single-nucleotide polymorphisms (SNPs), insertions or deletions (indels) of short sequences, recombination and gene conversion, segmental duplications, gross chromosomal rearrangements (GCRs), and ploidy alterations (Guillamón and Barrio 2017). SNPs can occur in coding and noncoding regions. In noncoding regions, SNPs mostly affect transcription (Guillamón and Barrio 2017). For example, an experimental evolution or UV-induction study in yeast identified causal mutations for acquired acetic acid tolerance in the genes *ASG1*, *ADH3*, and *SKS1* (González-Ramos et al. 2016). Another study demonstrated the development of multi-stress tolerance after the evolution of *S. cerevisiae* strains to improve acetic acid tolerance. The evolved strain maintained its redox homeostasis under oxidative stress, an ability attributed to enhanced catalase (CAT) and glutathione *S*-transferase (GST) activities (Gurdo et al. 2018). ﻿ *S. boulardii* strains that promoted high acetic acid production and antimicrobial properties presented SNPs on *sdh1*^F317Y^ and *whi2*^S287*^, as shown by whole-genome sequencing analysis (Offei et al. 2019).

Here, experimental evolution was used to study the genetic adaptive responses of *S. boulardii* yeast strains to acetic acid stress during fermentation. Whole-genome sequencing experiments, analysis of gene expression by quantitative real-time PCR, and RNA sequencing were performed in parallel to reveal candidate genes responsible for acetic acid stress adaptation. These approaches delivered a better understanding of the mechanisms for acetic acid tolerance in the less-explored yeast *S. boulardii*. The findings yielded targets for acetic acid tolerance improvement that may benefit a wide range of industrial applications.

## Materials and methods

### Strains and culture media

The yeast strains used for phenotypic analysis in this study were the wild-type *S. cerevisiae* strain BY4742 (*MAT*α *his3*Δ*1*; *leu2*Δ*0*; *lys2*Δ*0*; *ura3*Δ*0*) (Open Biosystems, Huntsville, AL, USA), *S. boulardii* Sb.P (van der Aa and Jespersen 2003), Enterol *S. boulardii*—a strain isolated from food supplement (CNCM I-745 commercial Enterol® strain, Biocodex, Warszawa, Poland), and evolutionary engineered strains of CNCM I-745 Enterol *S. boulardii* ev16 and ev17 (KMUTT, Bangkok, Thailand). The yeast strains were routinely grown in yeast extract-peptone-dextrose (YPD) medium containing 1% yeast extract, 2% Bacto peptone, and 2% dextrose. Meanwhile, the evolution experiment was performed in YPD with an increasing concentration of acetic acid, adjusted to pH 4.5 with HCl or 4 M KOH.

### Experimental adaptive laboratory evolution of* S. boulardii*

*S. boulardii* Sb.P and Ent were cultivated in a simple microaerobic system. Cells were propagated overnight in 3 mL of YPD medium and transferred to 500 mL of YPD in a 2-L Erlenmeyer flask at 30 °C for 2 days with shaking at 200 rpm. The cells were grown until the OD_600_ reached 2.0 in fermentation tubes containing 90 mL of YPD without or with acetic acid concentrations of 4.0, 6.0, 8.0, 10.0, and 12.0 g/L, adjusted to pH 4.5 with HCl and 4 M KOH. Then, 7.0 g/L and 5.0 g/L acetic acid were used in the evolution experiment for Ent and Sb.P, respectively. Cultures containing acetic acid were inoculated by transferring 3 mL directly from the previous YPD medium without acetic acid. Inoculation of cultures without acetic acid was performed by collecting cells from 3 mL of broth from the previous culture with acetic acid, washing the cells with sterile water and transferring them to YPD without pH adjustment. During the 12 cycles of experimental evolution of the yeast cells, fermentation progress was assessed by following weight loss as a measure for yeast fermentative activity.

### Screening and stabilization of acetic acid tolerance

After 12 cycles, the evolved cells were plated on YPD agar and incubated at 37 °C for 1–2 days. Random single colonies were cultured in 24-well YPD plates for 2 days and transferred to new 96-well YPD plates for evaluation of growth in YPD supplemented with acetic acid. The optical density was recorded every 10 min using a Multiskan™ FC Microplate Photometer (Thermo Fisher Scientific Inc., Waltham, USA).

### Determination of acetic acid stress sensitivity of *S. cerevisiae* strains

The acetic acid tolerances of the evolved yeast strains were tested by spot dilution assay on solid media containing different acetic acid concentrations, along with the ancestral *S. boulardii* strains. Cells were cultured in YPD at 30 °C for 18 h, harvested, and resuspended in distilled water at the same cell concentration (OD_600_ of 0.1). The cell suspension was diluted serially 10-fold (10^−1^–10^−4^). Then, 3 μL was spotted onto YPD plates supplemented with different concentrations of acetic acid (4.0, 6.0, 8.0, and 10.0 g/L) at pH 4.5. The growth of the yeast cells was assessed after incubation at 37 °C for 2–3 days.

### Determination of cell growth and cell survival under acetic acid stress

*S. boulardii* Ent (ancestral) and evolved *S. boulardii* (Ent strains ev16 and ev17) were routinely grown in YPD. These strains were precultured at 30 °C overnight with shaking. Aliquots of cells were then used to inoculate fresh YPD and regrown at 37 °C with shaking to an OD_600_ of 0.6. Then, the culture was split, and the cells were washed with sterile water. Cells were transferred to fresh YPD with and without a 6.0 g/L final concentration of acetic acid, and both cultures were adjusted to a pH of 4.5. Culture samples were taken at 0, 2, 4, 6, 24, and 48 h to measure the OD_600_ by spectrophotometry to construct growth curves. In parallel, cells collected at each time point were plated on YPD agar and incubated at 30 °C for 2 days to determine cell survival by colony forming unit (CFU) count. At least two independent experiments were performed in triplicate. The adaptive ability and growth of evolved *S. boulardii* Ent ev7, ev4.3, ev4.1, evS5, and ev9.16, along with ev16 and ev17, were investigated. These strains were precultured at 30 °C overnight with shaking. Next, the cells were regrown in fresh YPD at 37 °C with shaking to an OD_600_ of 0.6. Then, the culture was split, and the cells were washed with sterile water. The yeasts were cultured in 96-well plates containing YPD with and without a 7.0 g/L final concentration of acetic acid. Both cultures were adjusted to a pH of 4.5. Then, the growth was observed by a Multiskan Sky Microplate Spectrophotometer (Thermo Fisher Scientific, Carlsbad, CA, USA). Phenotypic analysis was conducted by the spot assay. These cells were routinely cultured in YPD for 18 h and then adjusted to a concentration OD_600_ of 0.1. The cell suspension was diluted serially 10-fold (10^−1^–10^−4^). Three microliters of cells was spotted onto YPD plates supplemented with different concentrations of acetic acid (4.0, 6.0, and 8.0 g/L) at pH 4.5. The growth of yeast cells was assessed after incubation at 37 °C for 2–3 days.

### Fermentation

The *S. cerevisiae* BY4742, *S. boulardii* Ent (ancestral), and evolved *S. boulardii* Ent (ev16 and ev17) strains were grown in 250-mL flasks with 25 mL of YPD broth overnight at 30 °C and 150 rpm. The cells were diluted to an OD_600_ of 1.0 and transferred to 250-mL Erlenmeyer flasks containing 50 mL of yeast extract-peptone (YP) broth supplemented with 200 g/L glucose. The flasks were incubated at 37 °C with shaking at 200 rpm. Cell samples (1.5 mL) were harvested at 0, 6, 24, 48, 72, and 96 h to determine growth by spectrophotometry and biomass by cell dry weight. The concentrations of glucose, ethanol, glycerol, and acetic acid in the broth media were determined by HPLC (Shimadzu, Kyoto, Japan) with an Aminex HPX-87H ion-exchange column (Bio-Rad, Hercules, CA, USA). A mobile phase of 5 mM H_2_SO_4_ was used at a flow rate of 0.6 mL min^−1^, with a column temperature of 65 °C.

### Whole-genome sequencing and mutation identification

Genomic DNA samples of Ent and the evolved Ent ev16 and ev17 were extracted using a PureLink™ Genomic DNA Mini Kit (Invitrogen™, Thermo Fisher Scientific, Carlsbad, CA, USA). Whole-genome sequencing of the DNA samples was performed using an Illumina HiSeq X Ten platform (Illumina, San Diego, CA, USA) with the pair-end 2 × 150 bp format. The sequence reads of each strain were mapped onto the reference genome of the probiotic yeast *S. boulardii* ATCC MYA-796 (GenBank: JRHY00000000) (Batista et al. 2014) using the Burrows–Wheeler alignment (BWA) tool (Li and Durbin 2009). Identification of single-nucleotide variants (SNVs) was performed using the Sequence Alignment/Map tool (Li et al. 2009). Trimmomatic was used to remove adapter sequences and low-quality reads from the raw reads to eliminate bias in the analysis (Bolger et al. 2014). Following the removal of duplicates using Sambamba and the identification of variants with SAM tools, information about each variant was acquired and classified by chromosomes or scaffolds (Tarasov et al. 2015). The variants were annotated with SnpEff (Ulintz et al. 2019). The “per base sequence quality” plot generated by FastQC was used to check the overall quality of the produced data. Mapped and raw data statistics of *S. boulardii* are in Supplementary Tables S1 and S2. A Phred quality score of 20, which reflects 99% filtered database calling accuracy, was applied in Supplementary Table S3. The SNP mutations in coding regions were further classified into functional groups via the Gene Functional Classification tool in DAVID version 6.8, https://david.ncifcrf.gov/gene2gene.jsp (Huang da et al. 2007, 2009). All resulting DNA sequence data have been made available in the NCBI database, with accession numbers SRR22824388 (Ent ancestral), SRR22682660 (Ent ev16), and SRR22805713 (Ent ev17).

### Determination of acetic acid stress tolerance of deletion mutant strains

All deletion yeast strains used in this study were isogenic to BY4742 (Brachmann et al. 1998). They were generated by a PCR-based gene deletion strategy to generate start-to-stop codon deletions and replaced with a KanMX module (Giaever and Nislow 2014). The growth of deletion strains (*oaf1*Δ, *dan4*Δ, *sla1*Δ, *end3*Δ, *pip2*Δ, or *air2*Δ) was evaluated by a spot dilution assay on solid media at different acetic acid concentrations to determine tolerance to acetic acid compared to the wild-type *S. cerevisiae* BY4742. Spot tests were performed as described previously (Samakkarn et al. 2021). Additional *S. cerevisiae* strains lacking a transporter (including the strains *vba5*Δ, *fre7*Δ, *fre5*Δ, *gal2*Δ, *hxt1*Δ, *fat3*Δ, *ypt53*Δ, *opt2*Δ, *ade17*Δ, *mup3*Δ, *oac1*Δ, *mmp1*Δ, *gcv2*Δ, *sul1*Δ, and *mup1*Δ) or an amino acid metabolic gene (including the strains *aro10*Δ, *thi4*Δ, *tat1*Δ, *aro9*Δ, *agx1*Δ, *car2*Δ, *fms1*Δ, *shm2*Δ, *met6*Δ, *gcv1*Δ, *met17*Δ, and *mht1*Δ) were cultured in YPD containing 6.0 g/L acetic acid for 48 h. These strains were tested for cell survival on YPD agar plates and incubated for 24 h at 30 °C.

### Identification of specific gene mutations

The evolved Ent strains were routinely cultured in YPD to observe the adaptive acetic acid stress response of *S. boulardii*, as previously described. Next, the evolved Ent strains ev7, ev4.3, ev4.1, evS5, and ev9.16, as well as the ancestral Ent strain, were selected to examine *PAN1* mutations by the polymerase chain reaction (PCR) amplification technique with the specific oligonucleotides F: CTCAAATTACTGGAGGCGGT and R: CGGAACCATATCCTCCGG, which cover the mutation site. PCR was performed with Q5 High-Fidelity DNA Polymerase (M0491, New England Biolabs, Ipswich, MA, USA), and the PCR products were checked by gel electrophoresis, purified, quantified by a NanoDrop™ One/OneC Microvolume UV–Vis spectrophotometer (Thermo Fisher Scientific Inc., Waltham, USA) and sent for sequencing (Macrogen Inc., Seoul, Korea). Sequence alignments were performed using Geneious Prime Java software version 11.0.14.1+1 (Kearse et al. 2012) (https://www.geneious.com/prime/).

### Gene induction and mRNA expression level analysis

The *S. boulardii* Ent (ancestral) and evolved Ent ev16 strains were grown overnight in YPD at 30 °C, diluted to an OD_600_ of 0.1 in fresh medium and regrown to an OD_600_ of approximately 0.6 at 37 °C. Next, the cells were washed twice using distilled water and transferred to YPD containing a final concentration of 6.0 g/L acetic acid adjusted to pH 4.5 for subsequent culturing for 1 h. The yeast cells were harvested and washed twice using distilled water and diethylpyrocarbonate (DEPC) water. Afterward, total RNA was extracted with the phenol–chloroform method (Schmitt et al. 1990) and purified using an RNeasy Mini Kit (Qiagen, Hilden, Germany) (Livak and Schmittgen 2001). cDNA synthesis and quantitative reverse transcription PCR (qRT-PCR) assays were performed as previously described (Samakkarn et al. 2021). The relative quantification of each transcript was calculated using the threshold cycle (2^−ΔΔCT^) method and normalized using the *ACT1* gene as an internal control. The primer sequences for qRT-PCR are listed in Supplementary Table S1.

### RNA sequencing and data analysis

Total RNA samples of the ancestral Ent and the evolved Ent ev16 strains under 6.0 g/L acetic acid, induced for 1 h at 37 °C, were used in this experiment. RNA-seq library preparation (Martin and Wang 2011) by using the Illumina TruSeq Stranded mRNA library and transcriptome sequencing were carried out as a service by Macrogen (Seoul, Korea). The reads were trimmed and mapped to the *S. boulardii* unique28 reference genome (https://www.ncbi.nlm.nih.gov/nuccore/LIOO00000000.1) (Khatri et al. 2017) using HISAT2 version 2.1.0, a splice-aware aligner (Kim et al. 2015) (https://ccb.jhu.edu/software/hisat2/index.shtml). The transcripts were assembled by StringTie version 2.1.3b (Pertea et al. 2015) (https://ccb.jhu.edu/software/stringtie/) with aligned reads. Expression profiles are represented as read count and normalization values, calculated based on transcript length and coverage depth. Normalization values were provided as FPKM (fragments per kilobase of transcript per million mapped reads)/RPKM (reads per kilobase of transcript per million mapped reads) and TPM (transcripts per kilobase million). Statistical analysis of the differentially expressed genes (DEGs) was performed using fold change, exact test using edgeR per comparison pair (Robinson et al. 2010) (https://rdrr.io/bioc/edgeR/man/exactTest.html). The significant results were selected on conditions of |fc| ≥ 2 and exact test raw *p* value < 0.05. Details can be found in Supplementary Fig. S1. All resulting RNA-seq data have been made available in the NCBI database, with accession numbers SRR22904015 (Ent ancestral) and SRR22904016 (Ent ev16).

### Statistical analysis

Data analysis was performed using SPSS Statistics for Mac v. 26 (IBM Corp., Armonk, NY). Student’s *t* test was applied. *P* values of 0.05 and 0.01 were considered statistically significant. At least two independent biological experiments were performed with at least three technical replicates.

## Results

### Adaptive laboratory evolution for constitutive acetic acid tolerance

**Fig. 1:**
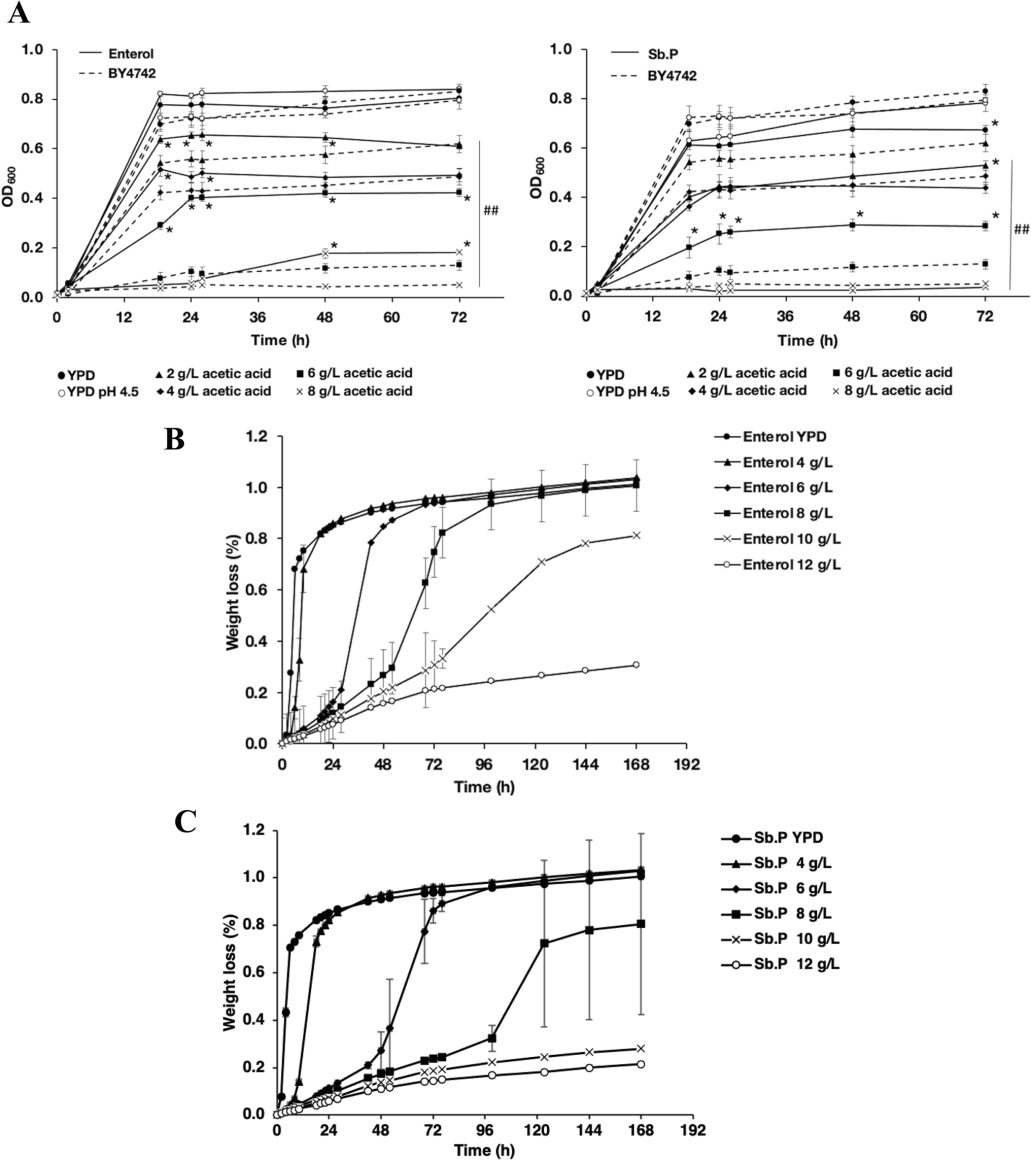
Growth (**A**) and fermentation performance (**B**, **C**) of *S. cerevisiae* and *S. boulardii* under acetic acid stress; **A** wild-type *S. cerevisiae* BY4742 and *S. boulardii* strains Ent and Sb.P were cultured in 96-well plates containing YPD without and with varying acetic acid concentrations (2, 4, 6, and 8 g/L) at pH 4.5 with shaking at 250 rpm; cell growth was determined using the Multiskan photometer; * and # indicate a significant difference, *p* < 0.05 and a two-tailed Student’s *t* test, respectively, based on comparisons with the wild-type *S. cerevisiae* BY4742 and the untreated condition; the fermentation performance of yeast *S. boulardii* was monitored by weight loss. Yeast strains **B** Ent and **C** Sb.P were evaluated under increasing acetic acid stress conditions; Ent and Sb.P were cultured in YPD and YPD supplemented with 4, 6, 8, 10, and 12 g/L acetic acid (pH 4.5)

First, the intrinsic tolerances of strains *S. cerevisiae* BY4742, *S. boulardii* Ent, and *S. boulardii* Sb.P to acetic acid were determined. Cells were grown on YPD medium at pH 4.5 without or with acetic acid (2–8 g/L). As shown, yeast cells were sensitive to increasing acetic acid concentrations (Fig. 1A). *S. cerevisiae* showed a dramatic decrease in growth at increased acetic acid concentrations in the range of 5–10 g/L. The *S. boulardii* Ent and Sb.P strains were more tolerant than *S. cerevisiae* BY4742 and tolerated high acetic acid concentration ranges of 7–10 g/L and 5–10 g/L, respectively (Fig. 1A). The *S. boulardii* strains outperformed *S. cerevisiae* BY4742, as demonstrated by their growth ability under acetic acid stress (Fig. 1A). Then, the fermentation performance of these strains was investigated under various acetic acid concentrations by monitoring the weight loss of the starter culture at various acetic acid concentrations (4 to 12 g/L). Both strains showed a gradual decrease in fermentation performance as the acetic acid concentrations increased. Interestingly, the Ent strain retained its ability to ferment in medium containing up to 10 g/L acetic acid. In contrast, the Sb.P strain was unable to ferment under these stress conditions. These results indicated that *S. boulardii* Ent was more tolerant to acetic acid than Sb.P (Fig. 1B, C). Based on the decrease in weight loss, the acetic acid stress tolerance thresholds for the Ent and Sb.P strains were determined to be 6 and 4 g/L acetic acid, respectively (Fig. 1B, C).

**Fig. 2:**
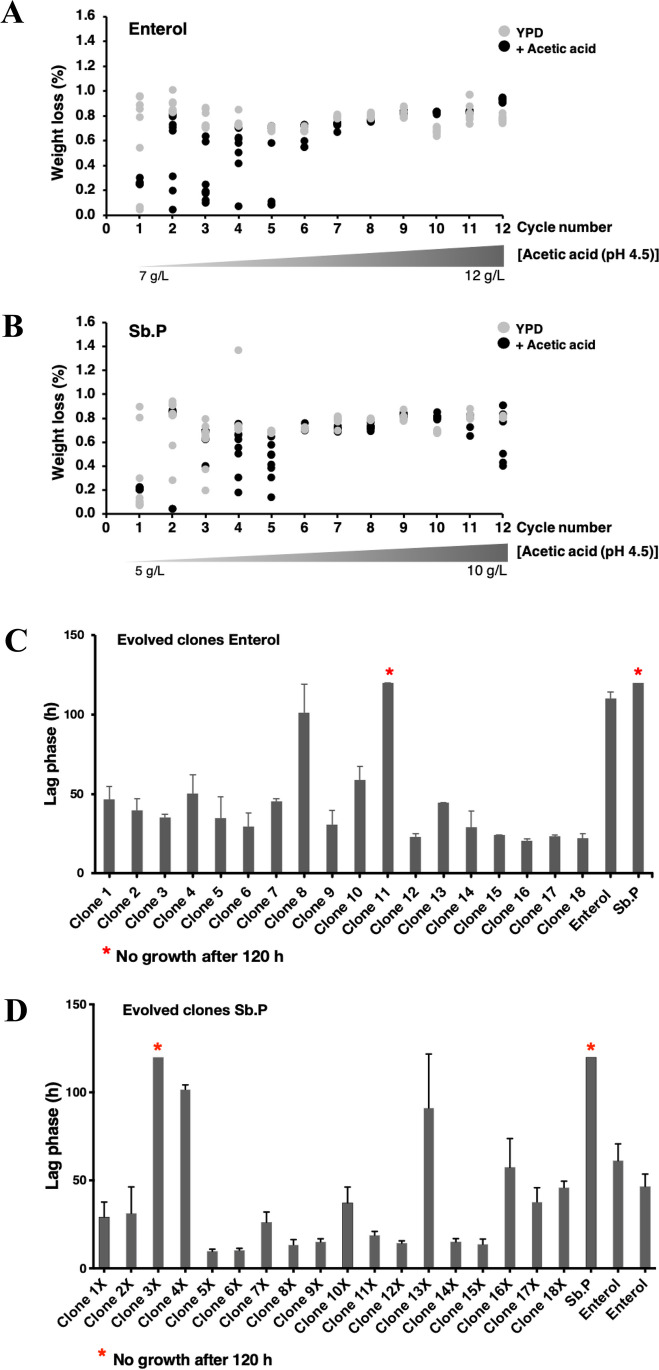
The evolution of *S. boulardii* under increasing acetic acid pressure. **A** The fermentation performance of Ent under slightly increasing concentrations (7–12 g/L) of acetic acid at pH 4.5 is presented in black dots, and after transfer to YPD without pH adjustment, it is presented in gray dots. **B** The fermentation performance of Sb.P under slightly increasing (5–10 g/L) acetic acid at pH 4.5 is presented in black dots, and that after transfer to YPD without pH adjustment is presented in gray dots; each strain was cultured in 10 tubes and monitored by the percentage of weight loss after 2 days. The adaptive abilities of evolved **C** Ent and **D** Sb.P strains at 7 and 4.5–5.0 g/L acetic acid, respectively, were monitored by the lag-phase time

Next, experimental evolution was performed as a means to gain insights into the genetic basis underlying the tolerance of *S. boulardii* to acetic acid stress. Based on the previously determined responses to acetic acid (Fig. 1C), prolonged cultures of the Ent and Sb.P strains in YPD under acetic acid concentrations ranging from 7 to 12 g/L and 5 to 10 g/L (pH 4.5) were performed over 12 cycles (Fig. 2A, B). After 3 days of cultivation, the fermentation tubes showed a weight loss of 0.3% for Ent at 7 g/L acetic acid and 0.2% for Sb.P at 5 g/L (Fig. 2A, B). These cells were transferred to a nonselective cycle in YPD, and weight loss increased to 0.8–1.0%. Hereafter, a second fermentation cycle under acetic acid stress was performed. Ent showed better adaptive tolerance to acetic acid than Sb.P during evolution; variations in their acetic acid tolerance were found within 1–5 cycles. Upon completing the experimental evolution, after 12 cycles of evolution, Ent showed a similar weight loss for fermentations under stressed and unstressed conditions (gray dot in Fig. 2A, B). However, the evolved Sb.P strain appeared to struggle to grow at 10 g/L acetic acid (Fig. 2B). Subsequently, growth in the presence of 4.5–5.0 or 7.0 g/L acetic acid at pH 4.5 was evaluated for single colonies obtained from the evolved Sb.P and Ent (Fig. 2C, D). After the evolution experiment, most clones were more tolerant to acetic acid than the ancestral strain, as observed by shortened lag phases during growth under acetic acid stress (Fig. 2C, D). The Ent ev16 and ev17 clones showed the shortest lag-phase time of 20 h under acetic acid-induced stress (7 g/L) compared to the lag phases of the ancestral Ent and Sb.P. strains of 100 h or longer, respectively (Fig. 2C). Likewise, most evolved Sb.P clones showed shortened lag phases under acetic acid stress (5 g/L). The Sb.P ev5X and ev6X clones showed a short lag-phase time of 12 h, indicating substantially faster growth than the ancestral strain (Fig. 2D).

**Fig. 3:**
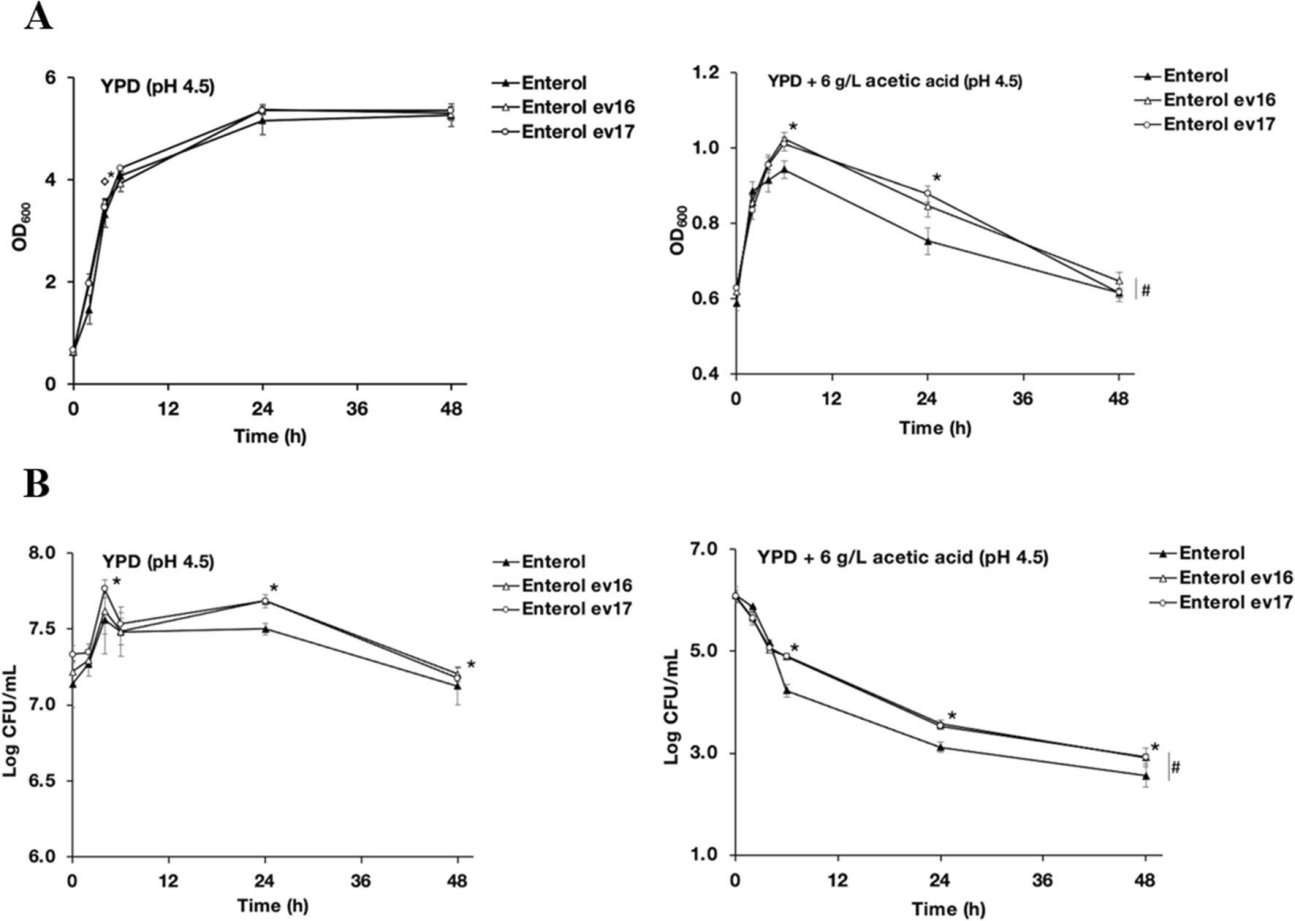
Growth of the *S. boulardii* ancestral Ent strain compared with the evolved strains Ent ev16 and Ent ev17 in acetic acid stress conditions; **A** the growth curves and **B** survival of the yeast strains under acetic acid stress; the ancestral Ent strain and the evolved *S. boulardii* Ent ev16 and ev17; strains were cultured in YPD medium or in YPD supplemented with 6 g/L acetic acid, adjusted to pH 4.5; error bars indicate standard deviations calculated from at least two independent experiments performed in triplicate; * indicates a significant difference, *p* < 0.05, two-tailed Student’s *t* test compared to the *S. boulardii* ancestor; # indicates a value of significant difference, *p* < 0.01, two-tailed Student’s *t* test compared to the untreated condition YPD at pH 4.5

Finally, the survival of the evolved yeast Ent clones Ent ev16 and ev17 was examined under acetic acid stress. Growth and CFU counts were evaluated in YPD with or without 6 g/L acetic acid, pH 4.5. The evolved Ent strains grew well and survived better than the ancestral strains at 6, 24, or 48 h (Fig. 3A, B). The selected clones of evolved *S. boulardii* strains could survive better than the ancestral strain under high acetic acid stress of 6 g/L and temperatures of 37 °C. The higher viability of the evolved strains illustrated their improved acid tolerance.

### Evolved *S. boulardii* displayed an altered metabolic profile

**Fig. 4:**
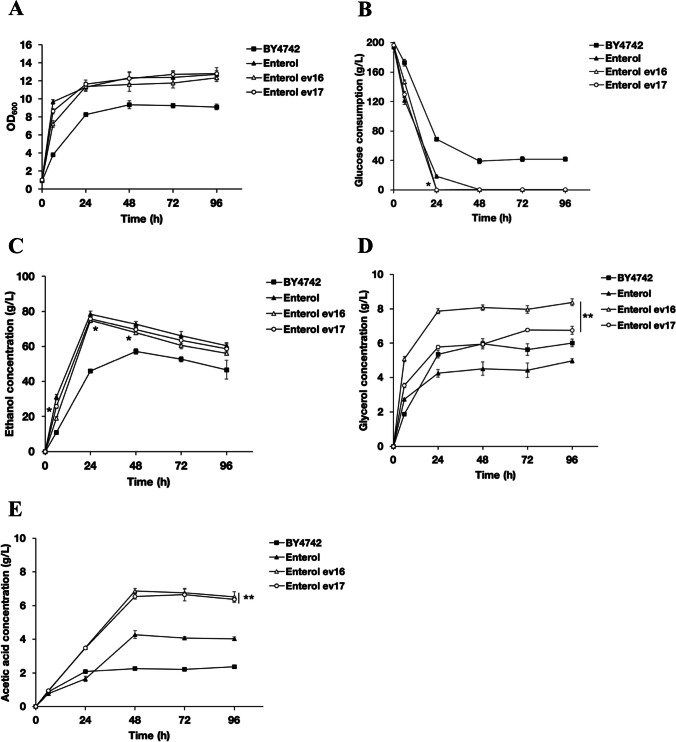
Fermentation profiles of *S. cerevisiae* BY4742, of *S. boulardii* ancestral strain Ent, and of evolved *S. boulardii* ev16, ev17 cultured at a high concentration of glucose (20% wt/vol) for 12–96 h at 37 °C; **A** cell growth was monitored (OD_600_) by a spectrophotometer; **B** yeast glucose consumption; **C** ethanol production; **D** glycerol production; **E** acetic acid production; the glucose, ethanol, glycerol, and acetic acid concentrations were calculated from three replicates; * indicates a significant difference, *p* < 0.05 two-tailed Student’s *t* test compared to the *S. boulardii* ancestor; ** indicates *p* < 0.01, two-tailed Student’s *t* test compared to the *S. boulardii* ancestor and significance of differences at all time points

The fermentation profiles of different yeast strains, including the *S. boulardii* ancestral Ent and evolved Ent strains Ent ev16 and ev17, are shown in Fig. 4. Glucose consumption and the production of metabolites, i.e., ethanol, glycerol, and acetic acid, were determined using starting glucose concentrations of 200 g/L as a substrate at 37 °C with shaking. The evolved Ent strains Ent ev16 and ev17 showed better growth at 37 °C than *S. cerevisiae* BY4742 (Fig. 4A, Table 1). In addition, the evolved Ent strains Ent ev16 and ev17 showed rapid glucose consumption, completing fermentation by 24 h (Fig. 4B, Table 1). Regarding ethanol production, the *S. boulardii* ancestral Ent (78.37 g/L) showed higher ethanol production than *S. cerevisiae* BY4742 (45.86 g/L) at 24 h (Fig. 4C, Table 1). Thus, *S. boulardii* displayed higher ethanol productivity than *S. cerevisiae* (Table 1). The evolved Ent strains Ent ev16 and ev17 produced slightly lower levels of ethanol (74.96 and 75.63 g/L) than the ancestral strain (Fig. 4C, Table 1).

**Table 1:** Fermentation and biomass profiles of *S. cerevisiae* and evolved *S. boulardii* strains, in YPD containing 20% glucose (w/v) at 24 h (top line) or 48 h (bottom line) of fermentation

Parameter^a^/strains	BY4742 (*S. cerevisiae*)	Enterol (*S. boulardii*)	Enterol ev16 (*S. boulardii*)	Enterol ev17 (*S. boulardii*)
Glucose consumption (g/L h^−1^)	5.52	7.30	8.17	8.18
	3.38	4.03	4.08	4.09
Glucose (g/L)	68.74 ± 2.38	18.64 ± 1.29	0.11 ± 0.02^*^	0.13 ± 0.01^*^
	39.12 ± 3.52	0.1 ± 0.01	0.26 ± 0.02^*^	0.21 ± 0.02^*^
Glycerol concentration (g/L)	5.36 ± 0.21	4.25 ± 0.22	7.86 ± 0.14^*^	5.77 ± 0.09^*^
	5.97 ± 0.30	4.52 ± 0.38	8.07 ± 0.16^*^	5.94 ± 0.17^*^
Change in glycerol concentration (%)	-	-	+ 84.94	+ 35.76
			+ 78.54	+ 31.42
Glycerol yield (g per g of consumed glucose)	0.040	0.024	0.040	0.029
	0.037	0.023	0.041	0.030
Glycerol productivity (g/L h^−1^)	0.223	0.177	0.328	0.240
	0.124	0.094	0.168	0.124
Glycerol productivity (g/L g^−1^ of biomass h^−1^)	0.119	0.069	0.124	0.091
	0.058	0.034	0.063	0.044
Ethanol concentration (g/L)	45.86 ± 0.68	78.37 ± 1.83	74.96 ± 1.35^*^	75.63 ± 1.14^*^
	57.10 ± 1.44	72.58 ± 1.58	67.92 ± 1.09^*^	69.65 ± 0.78^*^
Change in ethanol concentration (%)	-	-	− 4.35	− 3.50
			− 6.42	− 4.04
Ethanol yield (g per g of consumed glucose)	0.346	0.447	0.382	0.385
	0.352	0.375	0.347	0.355
Ethanol productivity (g/L h^−1^)	1.911	3.265	3.123	3.151
	1.190	1.512	1.415	1.451
Ethanol productivity (g/L g^−1^ of biomass h^−1^)	1.018	1.271	1.182	1.192
	0.560	0.539	0.533	0.520
Acetic acid concentration (g/L)	2.09 ± 0.02	1.64 ± 0.16	3.40 ± 0.06^*^	3.47 ± 0.04^*^
	2.26 ± 0.06	4.28 ± 0.23	6.86 ± 0.17^*^	6.55 ± 0.17^*^
Change in acetic acid concentration (%)	-	-	+ 107.32	+ 111.59
			+ 60.28	+ 53.04
Acetic acid yield (g per g of consumed glucose)	0.016	0.009	0.018	0.018
	0.014	0.022	0.035	0.033
Acetic acid productivity (g/L h^−1^)	0.087	0.068	0.146	0.145
	0.047	0.089	0.143	0.136
Acetic acid productivity (g/L g^−1^ of biomass)	0.046	0.027	0.055	0.055
	0.022	0.032	0.054	0.049
Initial dry cell weight (g/L)	0.21 ± 0.02	0.24 ± 0.02	0.23 ± 0.01	0.23 ± 0.01
Final dry cell weight (g/L)	1.88 ± 0.04	2.57 ± 0.11	2.64 ± 0.10	2.64 ± 0.10
	2.13 ± 0.10	2.81 ± 0.15	2.65 ± 0.17	2.79 ± 0.15
Change in cell biomass (%)	-	-	+ 2.72	+ 2.72
			− 5.69	− 0.71
Biomass yield (g per g of glucose)	0.014	0.015	0.013	0.013
	0.013	0.014	0.014	0.014
Specific growth rate (g/L h^−1^)	0.078	0.107	0.110	0.110
	0.044	0.058	0.055	0.058

^*^p < 0.05 compared to the ancestral Enterol strain of *S. boulardii*

^a^Parameter values are for strains grown in yeast extract-peptone-dextrose (YPD), containing 20% glucose (w/v), taken at start where noted. Results were obtained from at least two independent experiments performed in triplicate

Furthermore, the evolved strains Ent ev16 and Ent ev17 produced significantly higher levels of glycerol (7.86 and 5.77 g/L) than their ancestral strain Ent (4.25 g/L) and *S. cerevisiae* BY4742 (5.36 g/L) at 24 h of fermentation (Fig. 4D, Table 1). After 48–96 h, glycerol production generally increased, with maximal production in the medium at 48 h by the evolved strains Ent ev16 (8.07 g/L) and Ent ev17 (5.94 g/L) and the ancestral strain Ent (4.52 g/L) (Fig. 4D, Table 1). The Ent ev16 strain showed the highest glycerol production, with a productivity rate of 0.063 g/L g^−1^ of biomass/h (Table 1). The enhanced glycerol production by the evolved yeast strains was evident in the Ent strains of *S. boulardii*, suggesting an essential regulatory response of metabolic shift toward glycerol production during acid stress*.*

Moreover, the evolved *S. boulardii* strains also showed increased acetic acid production when compared to the ancestral strains (Fig. 4E, Table 1), while *S. cerevisiae* BY4742 showed the lowest acetic acid production (Fig. 4E). Interestingly, the Ent strains Ent ev16 and ev17 produced the highest levels of acetic acid at 6.86 and 6.55 g/L, and the ancestral strain produced 4.28 g/L at 48 h. Thus, the evolved yeast strains showed enhanced acetic acid production (Fig. 4E).

### Mutations associated with acetic acid tolerance of the evolved *S. boulardii*

To identify the adaptive mutations that enabled the evolved *S. boulardii* strains to tolerate acetic acid stress, whole-genome sequencing of the Ent ancestor and the two most tolerant evolved clones, Ent ev16 and Ent ev17, was performed. The sequenced genomes of these strains were compared with the reference *S. boulardii* strain ATCC MYA-796 (GenBank: JRHY00000000), which has a genome length of 12,001,065 bp (Supplementary Table S2). The sequencing reads were mapped onto the reference genome using the Burrows–Wheeler Alignment (BWA) alignment tool (Li and Durbin 2009) (http://bio-bwa.sourceforge.net). The *S. boulardii* sequences showed raw data and filtered data Q20 scores of 96% and 99%, respectively (Supplementary Tables S3 and S4). The percentages of mapped sites for the ancestral Ent, Ent ev16, and Ent ev17 were 99.71%, 99.71%, and 99.73%, respectively, indicating efficient mapping of *S. boulardii* Ent to the reference strain (Supplementary Table S2). The total reads for Ent (6,326,040), Ent ev16 (6,691,164), and Ent ev17 (6,927,114) (roughly 85–93% reads) were mapped, with average alignment depths of 62–73 reads (Supplementary Table S2). An average mapping depth of 50 reads is necessary to accurately determine the single-nucleotide variations (SNVs) and minor indels for over 95% of the genome (Ajay et al. 2011; Sims et al. 2014). The SNP counts for *S. boulardii*, the ancestral Ent, Ent ev16, and Ent ev17 revealed a total of 1942, 1898, and 1965 SNPs, respectively, when compared with the reference strain *S. boulardii* strain ATCC MYA-796 (Supplementary Table S5).

### Causal mutations linked to acetic acid tolerance in evolved *S. boulardii*

The evolved Ent ev16, Ent ev17, and ancestral Ent strains showed various SNPs, including frameshift insertions and deletions resulting in amino acid changes, when compared with the reference *S*. *cerevisiae* var. *boulardii* strain ATCC MYA-796 (Table 3). The polymorphisms of SNPs and indels were annotated to functional clusters using the Gene Functional Classification Tool in DAVID (Huang da et al. 2007, 2009). We found four clusters that were mostly involved in cell wall components, particularly glycosyl-phosphatidylinositol (GPI)-anchor, transcription factors and regulation systems, zinc or metal ion binding, and cell membrane according to the enrichment scores (Table 2). In comparison to the ancestral Ent, the evolved strains ev16 and ev17 showed some common and different mutations (Table 3). Four mutations were shared by both the ev16 and ev17 evolved strains, of which 2 were homozygous (Table 3). These were *oaf1*^Ser57Pro^ ﻿and *dan4*^Thr192del^ (homozygous), and *pan1*^Thr486_Ser487insThrGlyMetMetProGlnThr^, *pan1*^Gln1101_Pro1102insProThrGln^, and ﻿*thi13*^Thr332Ala^ (heterozygous) (Table 3). *OAF1* encodes an oleate-activated transcription factor (Luo et al. 1996), and *DAN4* encodes a cell wall mannoprotein (Abramova et al. 2001). *PAN1* is involved in the actin cytoskeleton-regulatory complex and endocytosis (Zeng et al. 2001). *THI13* encodes a 4-amino-5-hydroxymethyl-2-methylpyrimidine phosphate synthase that functions in thiamine biosynthesis, enabling vitamin B1 production (Rodríguez-Navarro et al. 2002). Since the enhanced acetic acid tolerance was unknown, spot tests and gene expression analysis under acetic acid stress were performed to investigate their functional contribution.

**Table 2:** Functional annotation of the SNPs identified on coding regions of *S. boulardii* Enterol and the evolved Enterol genomes

Function	Gene
Cluster 1	Enrichment score: 1.83*
Lipid moiety-binding region: GPI-anchor amidated asparagine, GPI anchor, cell wall organization, stress-induced protein SRP1/TIP1	*TIR4*, *TIR1*, *DAN4*, *YPS6*, *YPS1*, *BIT61*
Cluster 2	Enrichment score: 1.82*
Transcription regulation, DNA binding, positive regulation of transcription from RNA polymerase II promoter	*RDS1*, *PDR1*, *RPS5*, *ACE2*, *CBF1*, *OAF1*, *SWI1*, *RSF2*, *TAF1*, *THI3*, *HMS1*, *RTT106*, *HOG1*, *CDC39*, *HIR2*, *MSH5*, *MCM2*, *CHS5*, *CTK1*, *ELP4*
Cluster 3	Enrichment score: 1.82*
Zinc-finger, zinc ion binding, metal ion binding	*AIR2*, *MSL5*, *NFI1*, *RSF2*, *UBP14*, *TIS11*, *SWI1*, *MCM2*, *ACE2*, *RDS1*, *PDR1*, *OAF1*, *TAD1*, *GLT1*, *DLD1*, *PPH21*, *THI3*
Cluster 4	Enrichment score: 1.44*
Cell membrane, endocytosis, mating projection tip	*PAN1*, *SLA1*, *PAL1*, *CHS3*, *YPS1*, *YPS6*, *BIT61*, *DAN4*, *CHS5*, *SEC3*

*p-value < 0.05

**Table 3:** Overview of genotypic changes in the evolved Enterol obtained after the laboratory evolution experiments under simulated acetic acid stress

Strains	SNPs/indels ^a^	Description of protein function^b^
Evolved Enterol ev16, ev17, and Enterol ancestral (unevolved)	*chs3*^2121insA^, *chs5*^Ter552^	Chitin synthase-related; required for chitin synthesis of cell wall composition and populate a compartment in endocytic pathways
	*tir4*^His166fs^	Cell wall mannoprotein biosynthesis
	*ubp14*^304-7insT^	Ubiquitin-specific protease
	*rds1*^Gln695Lys^	Putative zinc cluster transcription factor involved in drug sensitivity response
	*cdc39*^Leu1572Phe^	Subunit of the CCR4-NOT1 core complex regulated multiple level of eukaryotic gene expression
	*pph21*^His169Asn^	Catalytic subunit of protein phosphatase 2A (PP2A) play important roles in signal transduction activities at cytoplasm
	*msh5*^Cys174Gly^	Protein of the MutS family involved in DNA alkylation tolerance
	*ctk1*^Tyr521dup^	Catalytic (alpha) subunit of C-terminal domain kinase I (CTDK-I) essential for translation process
	*msl5*^Ile316Met^	Component of the commitment complex; determines the initial step in the splicing process
	*yps1*^Asn480Asp^	Aspartic protease is a member of yapsin family of proteases involved in glycosylphosphatidylinositol (GPI) anchor activity
	*elp4*^Asn227Asp^	Subunit of hexameric RecA-like ATPase Elp456 elongator subcomplex
	*taf1*^Glu633_Glu635del^	TFIID subunit, involved in RNA pol II transcription initiation
	*bit61*^Leu516fs^	Subunit of TORC2 membrane-associated complex associated cell cycle and cell wall integrity
	*cbf1*^Met53fs^	Basic helix-loop-helix (bHLH) protein related DNA replication stress response
Evolved Enterol ev16	*sla1*^Ala838_Ser842del^	Cytoskeletal protein binding protein essential for actin regulation and endocytosis
	*rps5*^Pro514Thr^	Protein component of the small (40S) ribosomal subunit essential for viability
	*nfi1*^Asn18del^	SUMO E3 ligase associated nuclear pore complexes (NPCs) function
	*pal1*^His263Gln^	A member of EH domain proteins interact with Ede1p to be involved in endocytosis factor
	*air2*^Ile292fs^	RNA-binding subunit of the TRAMP nuclear RNA surveillance complex
Evolved Enterol ev17	*rsf2* ^*Gln317del*^	Zinc-finger protein regulator of nuclear and mitochondrial genes
	*swi1*^*Thr19del*^	Subunit of the SWI/SNF chromatin remodeling complex
	*rps5*^*Ala12Thr*^	Protein component of the small (40S) ribosomal subunit essential for viability
Evolved Enterol ev16 and Enterol ancestral (unevolved)	*mcm2*^Val347Ile^	DNA replication licensing factor in yeast and human
	*tad1*^Tyr241Asn and Thr517Arg^	tRNA-specific adenosine deaminase is related with the mammalian pre-mRNA editing enzymes Adar1/2
Evolved Enterol ev17 and Enterol ancestral (unevolved)	*glt1*^Asn321Asp^	NAD(+)-dependent glutamate synthase (GOGAT) for glutamate synthesis
	*dld1*^Leu203Val^	Major mitochondrial D-lactate dehydrogenase involved in lactic acid biosynthesis
	*sec3*^Ile1191Asn, Val1064Ala, and Asp849Glu^	Subunit of the exocyst complex via transported on actin cables
	*pdr1*^3056_3057insAAA in ev17^	Transcription factor regulates the pleiotropic drug response
	*hog1*^Leu302Ser^	Mitogen-activated protein kinase involved in osmoregulation
	*msl5*^Ala343Thr and Ser346Pro^	Component of the commitment complex related the initial step in the splicing process
	*yps6*^Arg35Lys^	Putative GPI-anchored aspartic protease relates protein degradation in environmental changes
	*ace2*^Arg294Pro^	Transcription factor required for septum destruction after cytokinesis and cell cycle
	*tis11*^Thr253Ile^	mRNA-binding protein, a member of the tristetraprolin family and related to iron stress
	*hir2*^Asp162His^	Subunit of HIR nucleosome assembly complex functions with SWI/SNF
	*hms1*^Gln385*^	bHLH protein exhibits myc-family transcription factor similarity
Evolved Enterol ev16 and Enterol ev17	*pan1*^Thr486_Ser487insThrGlyMetMetProGlnThr and Gln1101_Pro1102insProThrGln^	The EH-domain containing and part of actin cytoskeleton-regulatory complex Pan1-Sla1-End3 essential for endocytotic vesicles and endosome
	*dan4*^Thr192del^	Cell wall mannoprotein biosynthesis
	*oaf1*^Ser57Pro^	Oleate-activated transcription factor implicated in lipid metabolism
	*thi13*^thr332Ala^	A member of *THI5* family essential for the thiamine metabolism

^a^Indels were only indicated when they affected an open reading frame (ORF) and were non-synonymous when compared with the reference *Saccharomyces cerevisiae* var. *boulardii* strain ATCC MYA-796

^b^Comparative the data information of protein function based on yeast *S. cerevisiae*

**Fig. 5:**
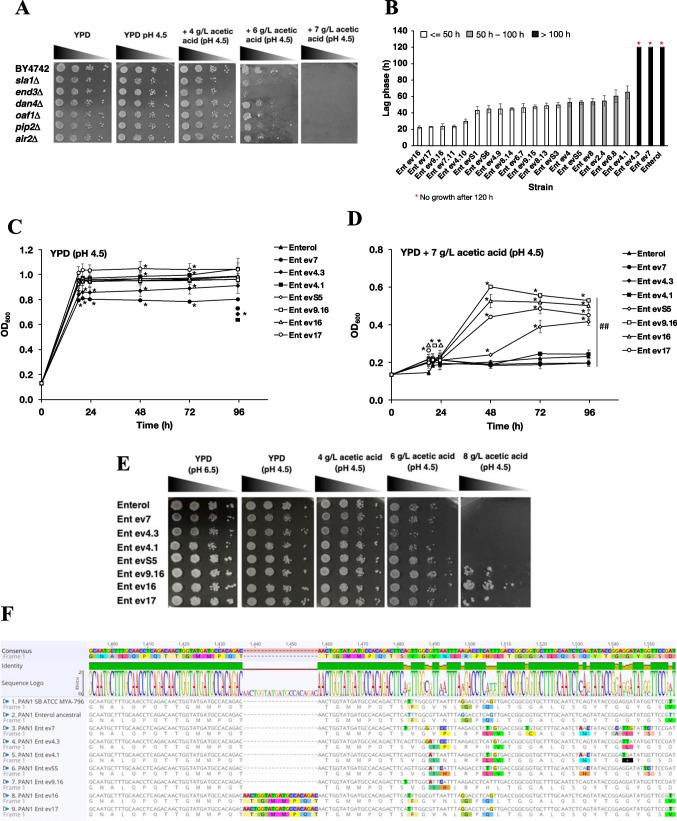

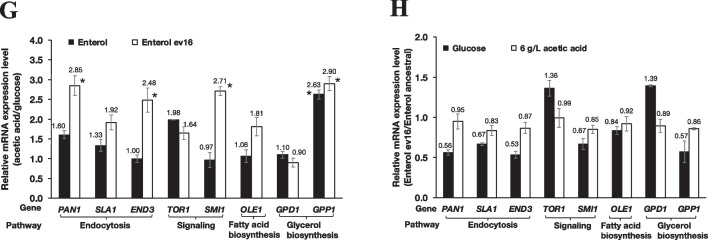
Effect of candidate gene mutations involved in acetic acid tolerance as obtained from the evolved Ent ev16 and ev17 strains; **A** phenotype analysis on acetic acid stress tolerance of deletion mutant strains; the wild-type *S. cerevisiae* BY4742 and strains lacking genes *sla1*, *end3*, *dan4*, *oaf1*, *pip2*, and *air2* were precultured in YPD liquid medium at 30 °C overnight with shaking; cells were serially diluted 10-fold (10^−1^ to 10^−4^), spotted on YPD plates supplemented with different concentrations of acetic acid (4, 6, and 7 g/L) at pH 4.5, and incubated at 30 °C for 2–5 days; **B** the adaptive abilities of various evolved Ent strains at 7 g/L acetic acid were monitored by the lag-phase time in cultivation; **C** the growth curves of various evolved yeast strains in YPD culture medium, pH 4.5; and **D** YPD culture medium supplemented with 7 g/L acetic acid, pH 4.5. * and ^##^ indicate significant differences, *p* < 0.05 and *p* < 0.01 and a two-tailed Student’s *t* test, respectively, based on comparisons with the wild-type *S. boulardii* Ent ancestral and the untreated condition; **E** phenotypic analysis of various evolved Ent strains presented on solid YPD media, pH 4.5, supplemented with 4.0, 6.0, and 8.0 g/L of acetic acid, respectively, was performed by a spot assay for 2–3 days; **F** genomic variation of the *PAN1* gene in the evolved Ent strains was observed by PCR performed with specific oligonucleotides (presented in the “Materials and methods” section), sequencing of obtained fragments, and subsequent sequence alignment; **G** relative mRNA expression levels of acetic acid stress response gene induction in the strain Ent ancestral and the evolved Ent ev16 *S. boulardii* strain grown in YPD medium containing 6 g/L acetic acid at pH 4.5 for 1 h versus YPD medium only; **H** relative mRNA expression level of the evolved Ent ev16 strain versus the Ent ancestral strain grown in YPD without or with 6 g/L acetic acid at pH 4.5 for 1 h; a relative expression level of 2-fold lower or higher was considered significant (*); error bars indicate standard deviations calculated from at least two independent experiments performed in triplicate; relative expression levels were obtained via the comparative CT method for the quantification of 2^−ΔΔCT^ values (Livak and Schmittgen 2001)

Notably, Pan1-Sla1-End3 is associated with the actin cytoskeleton-regulatory complex and promotes protein–protein interactions that are essential for endocytosis (Zeng et al. 2001). Therefore, these candidate genes were further evaluated. All the deletion strains lacking the *SLA1* or *END3* gene showed impaired growth under acetic acid stress compared to the wild-type *S. cerevisiae* BY4742 strain (Fig. 5A). Deletion strains lacking *PAN1* could not be investigated because *PAN1* has been shown to be essential for cell survival (Tang and Cai 1996). Additionally, the *air2*Δ, *oaf1*Δ, *pip2*Δ, and *dan4*Δ strains were included in the analysis and showed a higher sensitivity to acetic acid stress than the wild-type strain (Fig. 5A), suggesting their contribution to acetic acid tolerance.

### Acetic acid-induced expression of *PAN1* and related genes

As previously shown in Fig. 2C, where the adaptive ability of different evolved yeast strains was evaluated in the presence of 7 g/L acetic acid, Ent ev9.16 and ev7.11 strains displayed an equally short lag phase as the evolved Ent strains ev16 and ev17 (Fig. 5B). The second group of evolved Ent ev4, S5, ev8, ev2.4, ev6.8, and ev4.1 strains displayed a longer lag phase, approximately 45.1–65.1 h (Fig. 5B). The third group of evolved strains, ev4.3 and ev7, showed poor growth, similar to the ancestral Ent strain in both conditions, under YPD at pH 4.5 (Fig. 5C) and when supplemented with a final concentration of 7 g/L acetic acid (Fig. 5D). The evolved Ent ev17 (Ent ev17) reached a higher optical density faster than the ancestral Ent strain at 48 and 72 h under YPD conditions (Fig. 5C). Meanwhile, when compared to the Ent ancestral strain, the evolved Ent strains ev7 (Ent ev7) and ev4.3 (Ent ev4.3) showed poor growth (Fig. 5C). In YPD supplemented with 7 g/L acetic acid, the evolved Ent strains ev16 (Ent ev16) and ev17 (Ent ev17) grew faster than the Ent ancestral strain by approximately 18 h and the evolved Ent ev9.16 strain by 20 h, indicating that the evolved Ent strains ev16, ev17, and ev9.16 have the ability to quickly adapt to acetic acid stress (Fig. 5D). At 48 h, the evolved Ent evS5 (Ent evS5), ev16, ev17, and ev9.16 strains grew better than the Ent ancestral strain under acetic acid stress (Fig. 5D). To summarize, it was found that the evolved Ent strains developed better resistance to acid stress than the ancestral strain.

Furthermore, it was found that the evolved Ent evS5 strain started the log phase more slowly than the other Ent evolved strains, while the evolved Ent strains ev4.1, ev4.3, and ev7 were not different in growth when compared with the Ent ancestral strain (Fig. 5D). In addition, the evolved Ent ev4.1, ev4.3, and Ent ev7 strains and the Ent ancestral strain showed poor growth that was inhibited at 6 g/L acetic acid (Fig. 5E). In addition, the evolved Ent strains evS5, ev9.16, ev16, and ev17 exhibited impaired growth compared with the Ent ancestral strain. With increasing acetic acid stress, the evolved Ent strains ev9.16, ev16, and ev17 still persisted and thrived well compared to the Ent ancestral strain at 8 g/L acetic acid after 4 days of incubation (Fig. 5E).

Regulated transcription is another key process for cellular stress adaptation and tolerance in yeasts. The evolved Ent strain ev16 with increased acetic acid tolerance and high glycerol production (Fig. 4B–D and Table 1) was thus used to examine alterations in gene expression during metabolic adaptation to acetic acid stress. To identify gene mutations related to the observed increased acetic acid tolerance of strains Ent ev16 and ev17, the *PAN1*^Thr486_Ser487insThrGlyMetMetProGlnThr^ polymorphism was prioritized. Using Sanger sequencing, the sequence was verified in the Ent, Ent ev16, and Ent ev17 strains and in several other evolved clones. The genome of the probiotic yeast *S. boulardii* ATCC MYA-796 was again used as a reference for sequence comparison (Fig. 5F and Supplementary Fig. S1). The evolved Ent strains Ent ev16 and Ent ev17 displayed a duplication of the sequence AACTGGTATGATGCCACAGAC, leading to the insertion of amino acids ThrGlyMetMetProGlnThr at amino acid position 487 (P487), which was absent in the Ent ancestral strain (Fig. 5F, Table 3). Comparison of the adaptive growth ability of selected yeast Ent strains cultured under 7 g/L acetic acid for 12 cycles in the evolutionary experiment provided important observations. For example, the extra copy of duplicated ThrGlyMetMetProGlnThr was also identified in the Ent ev9.18, ev9.16, and ev7.11 strains, which displayed a shorter lag phase, similar to that of the ev16 and ev17 strains. However, this duplication was absent in the other group of the Ent strains, Ent ev4, evS5, ev8, ev2.4, ev6.8, ev4.1, and the Ent ancestral strain, with a longer lag phase and a high sensitivity (Fig. 5F, Table 3). When we examined other nonsynonymous mutations of the *PAN1* gene, the evolved Ent strains Ent ev16 and Ent ev17 displayed changes in amino acids at 11 positions compared to the Ent ancestral strain, including valine to phenylalanine (V/F), isoleucine to valine (I/V), histidine to asparagine (H/N), arginine to glycine (R/G), serine to proline (S/P), leucine to glutamine (L/Q), isoleucine to threonine (I/T), histidine to glutamine (H/Q), tryptophan to glycine (W/G), cysteine to glycine (C/G), and aspartic acid to valine (D/V) (Fig. 5F). However, these mutations were the same as those in the amino acid sequence of the probiotic yeast *S. boulardii* ATCC MYA-796 with low acetic acid tolerance. The other acetic acid-sensitive Ent strains Ent evS5, ev4.1, and ev4.3 showed different amino acid sequences compared to the reference strain ATCC MYA-769 but similar to that of the Ent ancestral strain, suggesting allelic differences in this diploid yeast. Some altered amino acids were found at seven positions for Ent ev7 (N to P, Q to L, L to V, G to C, Q to N, G to L, G to S), at five positions for Ent evS5 (S to P, L to H, I to T, W to G, and C to S), at six positions for Ent ev4.1 (H to N, S to P, L to V, I to T, H to N, and W to stop codon), and at three positions for Ent ev4.3 (V to I, N to P, and G to L) when compared to the Ent ancestral strain. However, these changes were similar to the amino acid sequence of the reference strain ATCC MYA-769 (Fig. 5F), which is sensitive to acetic acid. Thus, it is unlikely to be involved in conferring tolerance to acetic acid.

Regulated transcription is an important step in the cellular stress adaptation and tolerance of yeasts. Using gene expression analysis, we first evaluated the expression of genes with identified mutations according to the whole-genome analysis of the evolved Ent ev16 strain via quantitative reverse transcription PCR (qRT-PCR). We focused on genes in the pathways of endocytosis, signal transduction, and fatty acid and glycerol biosynthesis, as they were previously shown to be required for acid tolerance. The *SMI1* gene is essential for the cell wall integrity pathway, and the *OLE1* and *GPP1* genes are required for fatty acid and glycerol biosynthesis (Longo et al. 2022; Pahlman et al. 2001; Stukey et al. 1990). We observed increased expression levels of some genes involved in endocytic processes, such as *PAN1* and *END3*, cell wall integrity (*SMI1*), and glycerol biosynthesis (*GPP1*), under acetic acid treatment in Ent ev16, suggesting their involvement in the acetic acid response (Fig. 5G, H). Subsequently, qRT-PCR was performed for additional genes to observe changes in gene expression. The relative mRNA expression of *PAN1* and the related genes *SLA1*, *END3*, *TOR1*, *SMI1*, *OLE1*, *GPD1*, and *GPP1* were analyzed in comparison to those of the ancestral strain treated with 6 g/L acetic acid at pH 4.5. The expression of *PAN1* (2.85-fold), *SLA1* (1.92-fold), *END3* (2.48-fold), *SMI1* (2.71-fold), *OLE1* (1.81-fold), and *GPP1* (2.90-fold) was significantly upregulated in Ent ev16 following acetic acid treatment (Fig. 5G). This suggested the induction of *PAN1*, *SLA1*, and *END3* endocytic genes in response to acetic acid stress. However, only *GPP1* expression was significantly induced in both the Ent ancestral strain and the evolved Ent ev16 (Fig. 5G). It is possible that the genetic mutation observed in the evolved Ent ev16 strain may instead alter the enzymatic activity without causing any changes in gene expression. The *pan1*^Thr486_Ser487insThrGlyMetMetProGlnThr^ and *pan1*^Gln1101_Pro1102insProThrGln^ mutations may lead to a gain of function of Pan1, which, for unknown reasons, may affect endocytosis and promote acetic acid stress adaptation in *S. boulardii*. However, further studies are required to evaluate how these variant alleles can confer improved acetic acid tolerance.

### Transcriptomic profile of the evolved *S. boulardii* Ent ev16 in response to acetic acid challenge

**Fig. 6:**
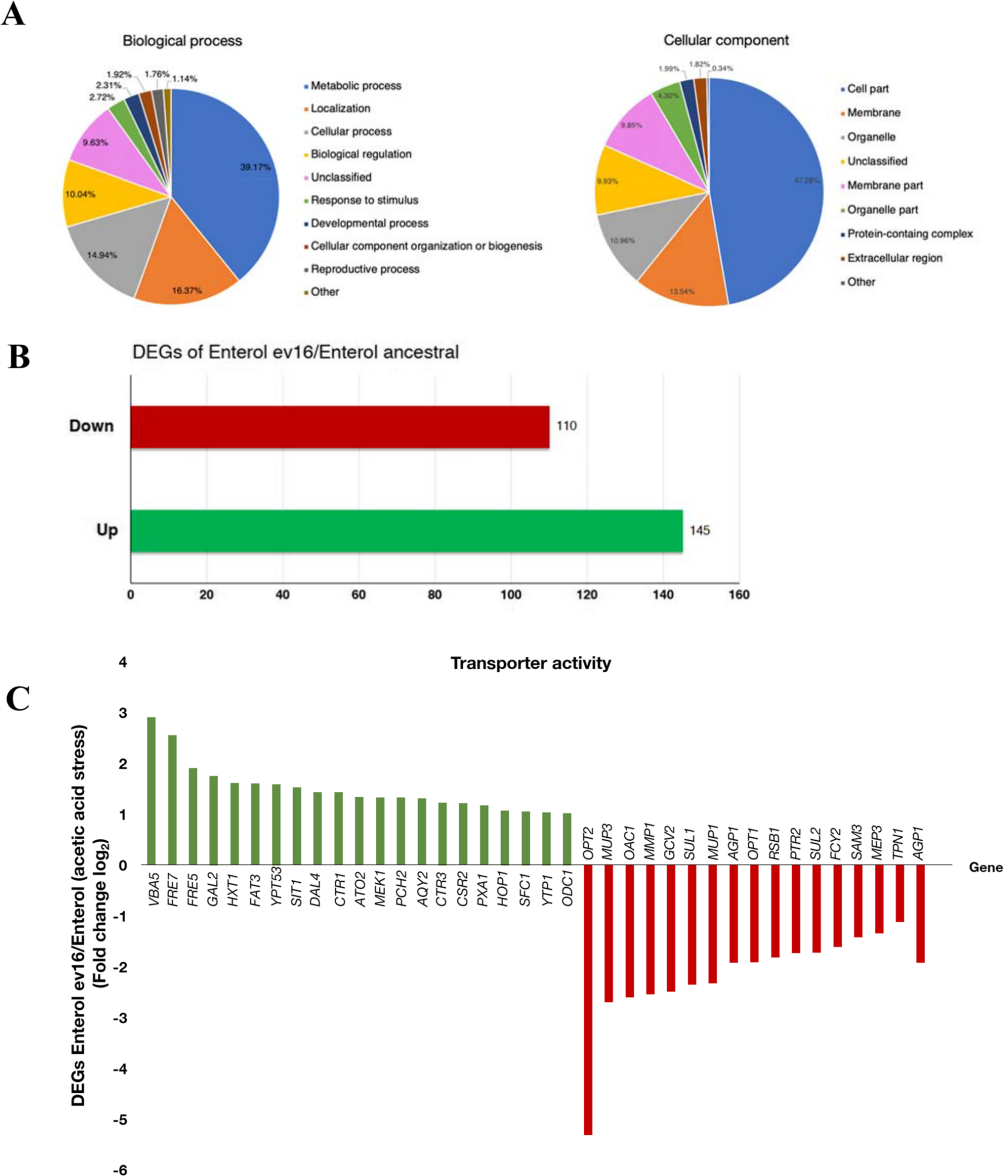

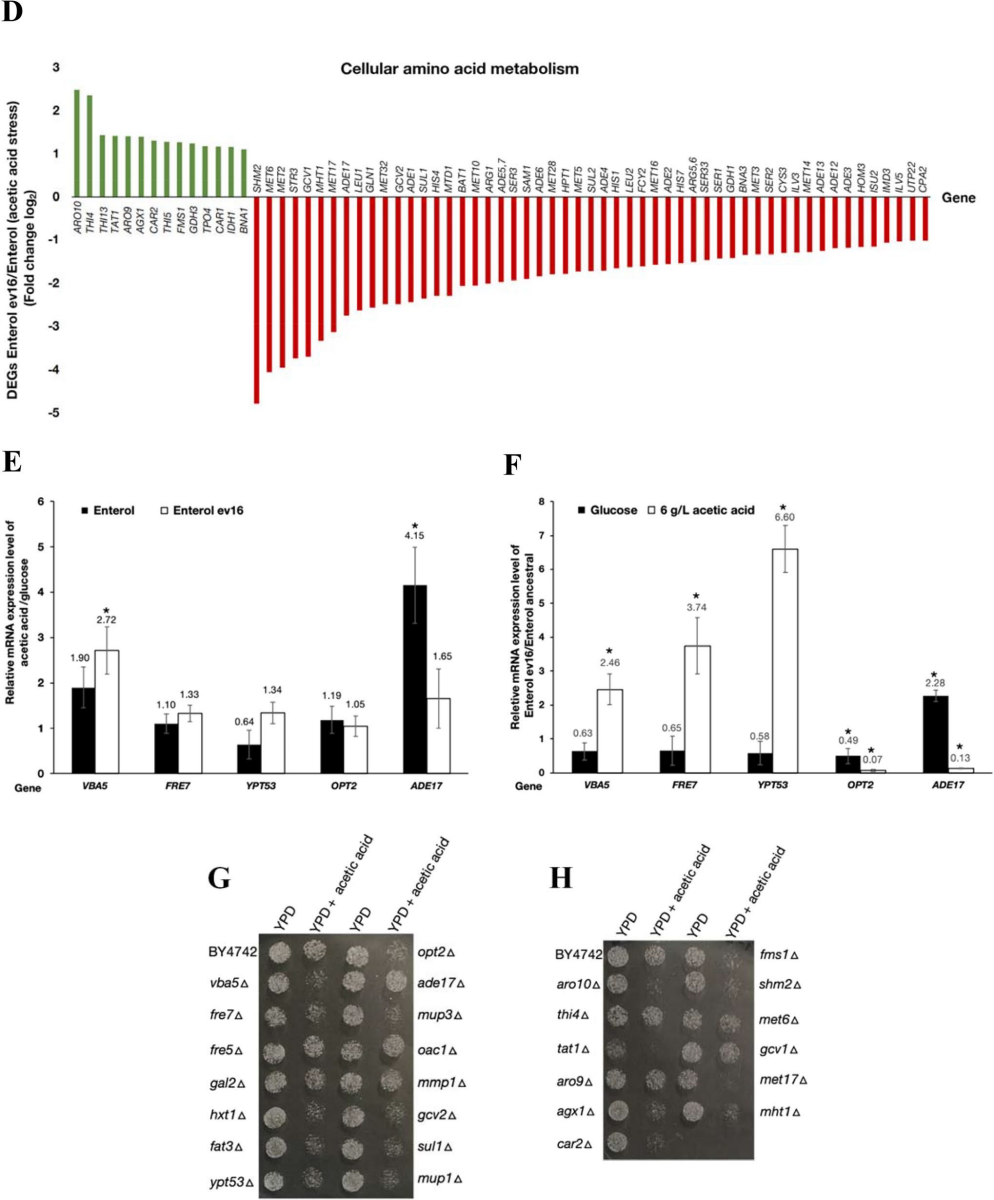
Transcriptomic profile of the ancestral Ent and the evolved Ent ev16 strain related with phenotypic effect of acetic acid stress; **A** gene expression levels of strain the evolved Ent ev16 versus the ancestral Ent strain were annotated by following the biological process and cellular component; **B** amount of the differentially expressed genes (DEGs) of the evolved Ent ev16 strain versus the ancestral Ent strain that presented fold changes (log_2_) ≥ 2 in significantly up- (green bar) or downregulated (red bar) genes, using two replicates with a statistical *p* value < 0.05; the DEG fold changes (log_2_) ≥ 2 of the evolved Ent ev16/the Ent ancestral strain presented genes related with transporter activity (**C**) and cellular amino acid metabolism (**D**), significantly up- (green bar) or downregulated (red bar); **E** relative mRNA expression levels of acetic acid stress response genes in the Ent (ancestral) and the evolved Ent ev16 *S. boulardii* strain grown in YPD medium containing medium with 6 g/L acetic acid at pH 4.5 for 1 h versus YPD medium only; **F** relative mRNA expression level of the evolved Ent ev16 versus the ancestral Ent strain grown in YPD without or with 6 g/L acetic acid at pH 4.5 for 1 h; a relative expression level of 2-fold lower or higher was considered significantly different (*); error bars indicate standard deviations calculated from at least two independent experiments performed in triplicate; relative expression levels were obtained via the comparative CT method for the quantification of 2^−ΔΔCT^ values; phenotype analysis on acetic acid stress tolerance of deletion mutant strains; the wild-type *S. cerevisiae* BY4742 and strains lacking some **G** transporter genes (*vba5*Δ, *fre7*Δ, *fre5*Δ, *gal2*Δ, *hxt1*Δ, *fat3*Δ, *ypt53*Δ, *opt2*Δ, *ade17*Δ, *mup3*Δ, *oac1*Δ, *mmp1*Δ, *gcv2*Δ, *sul1*Δ, and *mup1*Δ) and **H** amino acids (*aro10*Δ, *thi4*Δ, *tat1*Δ, *aro9*Δ, *agx1*Δ, *car2*Δ, *fms1*Δ, *shm2*Δ, *met6*Δ, *gcv1*Δ, *met17*Δ, and *mht1*Δ) were precultured in YPD liquid medium at 30 °C overnight with or without 6 g/L acetic acid at pH 4.5 for 48 h, and cells were diluted 10^−1^, spot cultured on YPD agar, and incubated for 24 h at 30 °C

We used a genome-wide transcriptomic approach via RNA sequencing analysis to determine the transcriptome of the evolved *S. cerevisiae* var. *boulardii* Ent ev16 strain. Since the evolved Ent ev16 strain displayed high acetic acid tolerance, its genome-wide transcriptional profile was examined and compared with that of the ancestral strain. Expression profiles were examined after exposure to 6 g/L acetic acid for 1 h. The RNA sequencing data showed the differentially expressed genes (DEGs) of the evolved Ent ev16 compared to the ancestral strain in the presence of acetic acid (Table 4 and Fig. 6C, D). Significant changes in gene expression were observed in gene-encoding plasma membrane transporters such as ATP-binding cassette (ABC) transporters, electron transporters, Golgi-to-endosome transporters, and major facilitator superfamily (MFS) transporters, including amino acid transporters. They are required to adjust the balance of various cellular metabolites to ensure cell survival at high acid levels. Moreover, changes in the expression of genes involved in amino acid metabolism were found in the Ent ev16 strain. The nitrogen metabolism of arginine, leucine, methionine, serine, and glutamine, which is required for energy production, including the tricarboxylic acid (TCA) cycle and redox balance, was repressed (Table 4). In contrast, the expression of genes involved in thiamine biosynthesis was upregulated in the evolved Ent ev16 strain (Fig. 6D and Table 4), promoting the synthesis of essential cofactor enzymes required for carbohydrate, amino acid, and lipid metabolism in this probiotic yeast. Thus, regulation of transport activity and amino acid metabolism are prime mechanisms in acetic acid stress adaptation and tolerance to promote cell survival in the evolved strains. In fact, the transcriptome expression pattern of Ent ev16 remarkably differed from that of the ancestral strain (Table 4). The DEGs were found to be significant for many genes involved in metabolic processes as well as cell membranes and organelles (Fig. 6A). The expression of 145 genes was upregulated and that of 110 genes was downregulated in the acetic acid-induced tolerant yeast Ent ev16 compared to its parental strain during acetic acid challenge (Fig. 6B).

**Table 4:** The expression levels of differentially expressed genes (DEGs) were selectively provided when compared between the evolved Enterol (Ent ev16) versus the ancestral Enterol strains under simulated acetic acid stress condition

Pathway/function	Protein_ID	Gene_Symbol	Description^a^	Ent_ev16/Ent. ancestral
Transporter	KQC42530.1	*VBA5*	Plasma membrane protein of the major facilitator superfamily (MFS)	7.483838
	KQC41276.1	*FRE7*	Putative ferric reductase with similarity to Fre2	5.847503
	KQC40802.1	*FRE5*	Putative ferric reductase with similarity to Fre2	3.741028
	KQC42203.1	*GAL2*	Galactose permease, transporter glucose	3.368527
	KQC41283.1	*HXT1*	Hexose transporter	3.051224
	KQC42795.1	*FAT3*	Fatty acid transporter	3.040469
	KQC41436.1	*YPT53*	Stress-induced Rab family GTPase, endocytosis, endosome	3.005306
	KQC44255.1	*SIT1*	Ferrioxamine B transporter ion transporter	2.877034
	KQC43183.1	*DAL4*	Allantoin permease	2.689628
	KQC40748.1	*CTR1*	High-affinity copper	2.687189
	KQC41351.1	*ATO2*	Putative transmembrane protein involved in export of ammonia	2.514019
	KQC40831.1	*MEK1*	Meiosis-specific serine/threonine protein kinase	2.510882
	KQC45588.1	*PCH2*	Hexameric ring ATPase that remodels chromosome axis protein Hop1	2.499508
	KQC40783.1	*AQY2*	Water channel protein	2.477214
	KQC42482.1	*CTR3*	High-affinity copper transporter of the plasma membrane	2.338267
	KQC40647.1	*CSR2*	Nuclear ubiquitin protein ligase binding protein	2.320455
	KQC40487.1	*PXA1*	Subunit of a heterodimeric peroxisomal ABC transport complex	2.243634
	KQC43278.1	*HOP1*	Meiosis-specific protein required for chromosome synapsis	2.099676
	KQC42889.1	*SFC1*	Mitochondrial succinate-fumarate transporter	2.075282
	KQC41556.1	*YTP1*	Putative type-III integral membrane protein	2.050415
	KQC40498.1	*ODC1*	Mitochondrial inner membrane transporter	2.025666
	KQC40785.1	*OPT2*	Oligopeptide transporter	− 39.569132
	KQC43610.1	*MUP3*	Low affinity methionine permease	− 6.479323
	KQC42738.1	*OAC1*	Mitochondrial inner membrane transporter	− 6.067879
	KQC42077.1	*MMP1*	High-affinity *S*-methylmethionine permease	− 5.812282
	KQC41939.1	*GCV2*	P subunit of the mitochondrial glycine decarboxylase complex	− 5.605879
	KQC45688.1	*SUL1*	High affinity sulfate permease of the SulP anion transporter family	− 5.108783
	KQC43830.1	*MUP1*	High affinity methionine permease	− 5.022137
	KQC45794.1	*AGP1*	Low-affinity amino acid permease with broad substrate range	− 3.788624
	KQC43158.1	*OPT1*	Proton-coupled oligopeptide transporter of the plasma membrane	− 3.751432
	KQC41096.1	*RSB1*	Suppressor of sphingoid LCB sensitivity of an LCB-lyase mutation	− 3.513485
	KQC42542.1	*PTR2*	Integral membrane	− 3.314776
	KQC42214.1	*SUL2*	High affinity sulfate permease	− 3.298349
	KQC44368.1	*FCY2*	Purine-cytosine permease	− 3.051087
	KQC40371.1	*SAM3*	High-affinity *S*-adenosylmethionine permease	− 2.672927
	KQC40737.1	*MEP3*	Ammonium permease of high capacity and low affinity	− 2.535364
	KQC44039.1	*TPN1*	Plasma membrane pyridoxine (vitamin B6) transporter	− 2.175493
Amino acid metabolism	KQC44620.1	*ARO10*	Phenylpyruvate decarboxylase	5.592195
	KQC43750.1	*THI4*	Thiazole synthase	5.078302
	KQC41642.1	*THI13*	Involved in synthesis of the thiamine precursor HMP	2.697496
	KQC45481.1	*TAT1*	Amino acid transporter for valine, leucine, isoleucine, and tyrosine	2.666198
	KQC43439.1	*ARO9*	Aromatic aminotransferase II	2.661068
	KQC44185.1	*AGX1*	Alanine: glyoxylate aminotransferase (AGT)	2.640765
	KQC42505.1	*CAR2*	L-Ornithine transaminase (OTAse)	2.463674
	KQC43167.1	*THI5*	Thi5 involved in synthesis of the thiamine precursor HMP	2.418843
	KQC41785.1	*FMS1*	Polyamine oxidase	2.400546
	KQC45699.1	*GDH3*	NADP(+)-dependent glutamate dehydrogenase	2.364376
	KQC40899.1	*TPO4*	Polyamine transporter of the major facilitator superfamily	2.263244
	KQC45837.1	*CAR1*	Arginase, catabolizes arginine to ornithine and urea	2.240821
	KQC41387.1	*IDH1*	Subunit of mitochondrial NAD(+)-dependent isocitrate dehydrogenase	2.222288
	KQC42952.1	*BNA1*	3-Hydroxyanthranilic acid dioxygenase	2.139855
	KQC44563.1	*APT2*	Putative adenine phosphoribosyltransferase	1.071718
	KQC42182.1	*SHM2*	Cytosolic serine hydroxymethyltransferase	− 27.484018
	KQC44406.1	*MET6*	Cobalamin-independent methionine synthase	− 16.569965
	KQC41591.1	*MET2*	L-Homoserine-*O*-acetyltransferase	− 15.501314
	KQC44037.1	*STR3*	Peroxisomal cystathionine beta-lyase	− 13.347240
	KQC44949.1	*GCV1*	T subunit of the mitochondrial glycine decarboxylase complex	− 13.023096
	KQC42076.1	*MHT1*	*S*-Methylmethionine-homocysteine methyltransferase	− 10.109192
	KQC42387.1	*MET17*	*O*-Acetyl homoserine-*O*-acetyl serine sulfhydrylase	− 8.793585
	KQC41874.1	*ADE17*	Enzyme of 'de novo' purine biosynthesis	− 6.725308
	KQC43886.1	*LEU1*	Isopropylmalate isomerase	− 6.202529
	KQC40652.1	*GLN1*	Glutamine metabolism	− 5.941626
	KQC44733.1	*MET32*	Zinc-finger DNA-binding	− 5.608989
	KQC41939.1	*GCV2*	P subunit of the mitochondrial glycine decarboxylase complex	− 5.605879
	KQC45764.1	*ADE1*	*N*-Succinyl-5-aminoimidazole-4-carboxamide ribotide synthetase	− 5.423384
	KQC45688.1	*SUL1*	High affinity sulfate permease of the SulP anion transporter family	− 5.108783
	KQC45289.1	*HIS4*	Multifunctional enzyme containing phosphoribosyl-ATP pyrophosphatase	− 4.905653
	KQC42555.1	*MTD1*	NAD-dependent 5,10-methylenetetrahydrafolate dehydrogenase	− 4.889177
	KQC43370.1	*BAT1*	Mitochondrial branched-chain amino acid (BCAA) aminotransferase	− 4.162589
Amino acid metabolism	KQC44129.1	*MET10*	Subunit alpha of assimilatory sulfite reductase	− 4.158779
	KQC41190.1	*ARG1*	Arginosuccinate synthetase	− 4.021111
	KQC44079.1	*ADE5,7*	Enzyme of the “de novo” purine nucleotide biosynthetic pathway	− 3.923477
	KQC44396.1	*SER3*	3-Phosphoglycerate dehydrogenase serine and glycine biosynthesis	− 3.812040
	KQC42287.1	*SAM1*	*S*-Adenosylmethionine synthetase	− 3.749601
	KQC43824.1	*ADE6*	Formylglycinamidine-ribonucleotide (FGAM)-synthetase	− 3.583555
	KQC43193.1	*MET28*	bZIP transcriptional activator in the Cbf1-Met4-Met28 complex	− 3.467680
	KQC44601.1	*HPT1*	Dimeric hypoxanthine-guanine phosphoribosyltransferase	− 3.449826
	KQC42850.1	*MET5*	Sulfite reductase beta subunit	− 3.313221
	KQC42214.1	*SUL2*	High affinity sulfate permease	− 3.298349
	KQC42045.1	*ADE4*	Phosphoribosylpyrophosphate amidotransferase (PRPPAT)	− 3.279341
	KQC44367.1	*HIS1*	ATP phosphoribosyltransferase in histidine biosynthesis	− 3.158937
	KQC45281.1	*LEU2*	Beta-isopropylmalate dehydrogenase (IMDH) in leucine biosynthesis	− 3.084144
	KQC44368.1	*FCY2*	Purine-cytosine permease	− 3.051087
	KQC40760.1	*MET16*	3'-Phosphoadenylsulfate reductase	− 2.970361
	KQC41028.1	*ADE2*	Phosphoribosylaminoimidazole carboxylase	− 2.938234
	KQC45644.1	*HIS7*	Imidazole glycerol phosphate synthase, glutamine amidotransferase	− 2.907885
	KQC44383.1	*ARG5,6*	Acetylglutamate kinase and *N*-acetyl-gamma-glutamyl-phosphate reductase in arginine biosynthesis	− 2.852658
	KQC43280.1	*SER33*	3-Phosphoglycerate dehydrogenase	− 2.752078
	KQC40978.1	*SER1*	3-Phosphoserine aminotransferase	− 2.688904
	KQC40809.1	*GDH1*	NADP(+)-dependent glutamate dehydrogenase	− 2.661146
	KQC43024.1	*BNA3*	Kynurenine aminotransferase, nicotinic acid biosynthesis (vitamin B3)	− 2.529246
	KQC42964.1	*MET3*	ATP sulfurylase in methionine biosynthesis	− 2.521497
	KQC43693.1	*SER2*	Phosphoserine phosphatase of the phosphoglycerate pathway	− 2.518280
	KQC45748.1	*CYS3*	Cystathionine gamma-lyase	− 2.469320
	KQC42958.1	*ILV3*	Dihydroxyacid dehydratase	− 2.452803
	KQC42630.1	*MET14*	Adenylylsulfate kinase	− 2.441374
	KQC42436.1	*ADE13*	Adenylosuccinate lyase	− 2.394539
	KQC41544.1	*ADE12*	Adenylosuccinate synthase	− 2.292804
	KQC43697.1	*ADE3*	Cytoplasmic trifunctional enzyme C1-tetrahydrofolate synthase	− 2.278842
	KQC44364.1	*HOM3*	Aspartate kinase (L-aspartate 4-P-transferase)	− 2.241280
	KQC40941.1	*ISU2*	Mitochondrial protein required for iron-sulfur protein synthesis	− 2.224529
	KQC42500.1	*IMD3*	Inosine monophosphate dehydrogenase	− 2.097239
	KQC42433.1	*ILV5*	Acetohydroxyacid reductoisomerase and mtDNA binding protein	− 2.058740
	KQC43799.1	*UTP22*	Component of the small-subunit processome	− 2.030159
Oxidative stress response	KQC41291.1	*YNR064C*	Epoxide hydrolase and detoxification	6.546002
	KQC44626.1	*PHO92*	Posttranscriptional regulator for phosphate metabolism	6.004980
	KQC40464.1	*OYE3*	Conserved NADPH oxidoreductase containing flavin mononucleotide	5.161394
	KQC42529.1	*GEX1*	Proton: glutathione antiporter	4.587540
	KQC42256.1	*TIS11*	mRNA-binding protein expressed during iron starvation	3.371556
	KQC42708.1	*SRX1*	Sulfiredoxin	3.304979
	KQC44553.1	*TSA2*	Stress inducible cytoplasmic thioredoxin peroxidase	2.940717
	KQC41999.1	*GTO1*	Omega-class glutathione transferase	2.708086
	KQC43301.1	*XBP1*	Transcriptional repressor	2.419911
	KQC43166.1	*AAD4*	Putative aryl-alcohol dehydrogenase	2.418601
	KQC40987.1	*DCS2*	m(7) GpppX pyrophosphatase regulator	2.339470
	KQC43982.1	*GPG1*	Putative gamma subunit of the heterotrimeric G protein	2.309664
	KQC44764.1	*SPR28*	Sporulation-specific protein	2.279524
	KQC44483.1	*HSP31*	Methylglyoxalase that converts methylglyoxal to D-lactate	2.135058
	KQC44353.1	*MXR1*	Methionine-*S*-sulfoxide reductase	− 2.665609
	KQC44387.1	*ALD5*	Mitochondrial aldehyde dehydrogenase involved acetate formation	− 2.565316
	KQC44058.1	*MIG2*	Zinc finger transcriptional repressor in low glucose induction	− 2.264420
Lipid and fatty acid metabolism/peroxisome	KQC43355.1	*POT1*	3-Ketoacyl-CoA thiolase with broad chain length specificity	4.232534
	KQC41180.1	*LDS2*	Protein involved in spore wall assembly; localizes to lipid droplets found on or outside of the prospore membrane	3.953611
	KQC42831.1	*PDX1*	E3-binding protein of the mitochondrial pyruvate dehydrogenase	3.767327
	KQC41528.1	*SPS19*	Peroxisomal 2,4-dienoyl-CoA reductase	3.241872
	KQC43065.1	*MEF2*	Mitochondrial elongation factor	3.024458
	KQC44730.1	*CTA1*	Catalase A for fatty acid beta oxidation	2.774603
	KQC45127.1	*INH1*	ATP hydrolysis inhibition by the F1F0-ATP synthase	2.719473
	KQC42621.1	*FOX2*	3-Hydroxyacyl-CoA dehydrogenase and enoyl-CoA hydratase	2.654284
	KQC42371.1	*ECI1*	Peroxisomal delta3, delta2-enoyl-CoA isomerase in lipid metabolic	2.645967
	KQC44054.1	*POX1*	Fatty-acyl coenzyme A oxidase in lipid metabolic process	2.581177
	KQC40624.1	*PDH1*	Putative 2-methylcitrate dehydratase in lipid metabolic process	2.436150
	KQC40536.1	*EHT1*	Acyl-coenzymeA:ethanol *O*-acyltransferase in lipid metabolic process	2.258347
	KQC43780.1	*CLD1*	Mitochondrial cardiolipin-specific phospholipase lipid metabolic process	2.240489
	KQC41872.1	*SHH3*	Putative mitochondrial inner membrane protein	2.079354
	KQC43369.1	*CRG1*	*S*-AdoMet-dependent methyltransferase involved in lipid homeostasis	2.073550
	KQC42388.1	*ACO1*	Aconitase in TCA cycle, localized in mitochondria	2.073256
	KQC42582.1	*YSR3*	Dihydrosphingosine 1-phosphate phosphatase in sphingolipid biosynthesis	2.051913
	KQC40498.1	*ODC1*	Mitochondrial inner membrane transporter	2.025666
	KQC42909.1	*OPI3*	Methylene-fatty-acyl-phospholipid synthase, lipid metabolic process	2.022791
	KQC42278.1	*UPS2*	Mitochondrial intermembrane space protein of phospholipid metabolism	2.021965
	KQC41731.1	*CAT2*	Carnitine acetyl-CoA transferase in mitochondria	2.000124
	KQC41096.1	*RSB1*	Suppressor of sphingoid LCB sensitivity of an LCB-lyase mutation	− 3.513485
	KQC44375.1	*CEM1*	Mitochondrial beta-keto-acyl synthase for fatty acid synthesis	− 3.141199
	KQC43149.1	*ACO2*	Putative mitochondrial aconitase isozyme in TCA cycle	− 2.015631
Cell wall synthesis and organization	KQC42392.1	*CHS3*	Chitin synthase III	8.621271
	KQC45318.1	*CHA1*	Catabolic L-serine (L-threonine) deaminase	8.488065
	KQC42299.1	*NCW2*	Hypothetical protein (structural constituent of the cell wall)	4.338859
	KQC42165.1	*AFB1*	MATalpha-specific a-factor blocker	3.356479
	KQC42773.1	*PIR3*	*O*-Glycosylated covalently-bound cell wall protein	3.322874
	KQC42420.1	*SPO77*	Meiosis-specific protein relevant in cell wall organization	3.270834
	KQC43002.1	*LOH1*	Hypothetical protein, cell wall organization or biosynthesis	3.001998
	KQC40666.1	*SMK1*	Middle sporulation-specific mitogen-activated protein kinase (MAPK)	2.815811
	KQC42485.1	*PUN1*	Plasma membrane protein with a role in cell wall integrity	2.386320
	KQC40647.1	*CSR2*	Nuclear ubiquitin protein ligase binding protein	2.320455
	KQC44921.1	*PST1*	Cell wall protein that contains a putative GPI-attachment site	2.131178
	KQC44412.1	*SHC1*	Sporulation-specific activator of Chs3 (chitin synthase III)	2.082493
	KQC44101.1	*YPS5*	Yps5 with similarity to GPI-anchored aspartic protease	2.002981
	KQC40927.1	*SSP2*	Sporulation specific protein that localizes to the spore wall	− 3.141633
	KQC41222.1	*SPO73*	Meiosis-specific protein	− 2.173651

a: the description is obtained from *Saccharomyces* Genome Database (SGD) at https://www.yeastgenome.org

### Abundance of transporter gene DEGs in acetic acid resistance

At least 37 genes involved in transporter transmembrane activity showed higher DEG abundances for the acetic acid-resistant clone (Fig. 6C and Table 4). Under this category, 21 upregulated genes were identified, according to their encoded transporter activities, as plasma membrane transporters belonging to the major facilitator superfamily (MFS), including the amino acid exporters for lysine and arginine (*VBA5*), ion transporters (*FRE5*, *FRE7*, *SIT1*, *CTR1*, *CTR3*, *ATO2*, and *SFC1*), ABC transport complex (*PXA1*), electron transporter (*YTP1*), mitochondrial inner membrane transporter (*ODC1*), galactose transporter (*GAL2*), hexose transporter (*HXT1*), fatty acid transporter (*FAT3*), allantoin oligopeptide transporter (*DAL4*), water transmembrane transporter (*AQY2*), and Golgi-to-endosome transporters (*YPT53* and *CSR2*) (Fig. 6C and Table 4).

Moreover, the evolved Ent ev16 also showed upregulated expression of signal-transducing genes (*MEK1*, *PCH2*, and *HOP1*) (Fig. 6C and Table 4). The AAA+ ATPase Pch2 is involved in the meiotic checkpoint. It is involved in the phosphorylation of the Hop1 axial component after the kinase Mek1 activation (Herruzo et al. 2021). Accordingly, meiotic induction can be passed to a daughter cell, embodying a form of epigenetic inheritance after being stimulated by environmental stress as cellular memory (Gutierrez et al. 2018). Thus, the activation of these meiotic genes in evolutionary Ent ev16 cells may promote acetic acid tolerance. In contrast, DEG analysis showed significant downregulated expression of the *PTR2*, *OPT2*, and *OPT1* genes, encoding oligopeptide importers such as di-, tri-, and tetrapeptides as well as glutathione (Becerra-Rodríguez et al. 2020), suggesting repression in Ent ev16.

We employed qRT-PCR analysis to determine the relative mRNA expression during induction with 6 g/L acetic acid for selected genes. Acetic acid induced the expression of the *VBA5* and *ADE17* genes in the Ent ev16 strain and the ancestral strain (Fig. 6E). In the Ent ev16 strain, upregulation of *VBA5*, *FRE7*, and *YPT53* and downregulation of *OPT2* and *ADE17* were found (Fig. 6F), which confirms the results of the DEG analysis of RNA-Seq. In addition, products of the downregulated *MUP3* and *MUP1* genes belong to the amino acid-polyamine-organocation (APC) superfamily of secondary carrier proteins, and their downregulation inhibits the importation of amino acids such as methionine and cysteine into the cytosol (Bianchi et al. 2019). The expression of the amino acid permease genes *AGP1*, *SAM3*, and *MMP1* was downregulated in the evolved Ent ev16 strain (Table 4 and Fig. 6C). Their products belong to the APC family of amino acid transporters. The evolved Ent ev16 may restrict broad ranges of amino acid uptake for certain types of amino acids, such as isoleucine, leucine, phenylalanine, cysteine, glutamine, asparagine, *S*-adenosylmethionine, and *S*-methylmethionine. Moreover, the expression levels of *SUL1*, *SUL2*, *FCY2*, and *MEP3* genes of the permease family and *TPN1* of the vitamin B6 transporter were also downregulated in the evolved Ent ev16 when compared with the ancestral strain under acetic acid stress (Table 4 and Fig. 6C, D). Similarly, the expression of *OAC1* and *RSB1*, encoding an oxaloacetate carrier and a lipid transporter that are localized to the mitochondrial and plasma membrane, respectively, was also downregulated (Table 4 and Fig. 6C).

### Abundance of amino acid metabolism DEGs in acetic acid resistance

Interestingly, dramatic changes in gene expression during acetic acid challenge were found in the cellular amino acid metabolism of *S. boulardii* (Table 4 and Fig. 6D). As previously reported for mitochondrial proteomics, the *Zygosaccharomyces bailii*-derived hybrid yeast-tolerant strain demonstrates increased amino acid metabolism of protein frequency percentage in the presence of acetic acid (Guerreiro et al. 2016). The DEGs of the evolved Ent ev16 versus the ancestral Ent strain showed 14 up- and 53 downregulated genes involved in amino acid metabolism (Fig. 6D and Table 4). Surprisingly, the maximum fold-change was found for *ARO10* (5.59-fold) and *ARO9* (2.66-fold) (Table 4). Both genes play a role in the Ehrlich pathway, where they are involved with the related aromatic aminotransferase Aro8 in the production of L-phenylalanine (Iraqui et al. 1998). Induction of the aromatic amino acid genes *ARO9* and *ARO10* is stimulated by a poor nitrogen source or upon TORC1 inhibition (Lee and Hahn 2013). *ARO9* expression is regulated by general control of amino acid biosynthesis. Yeast cells can use aromatic amino acids as a sole nitrogen source. Thus, the increased *ARO* gene expression in the Ent ev16 strain may promote nitrogen use, which is necessary for cell growth under acetic acid stress, as shown by the deletion of *ARO10*, which reduced cell survival under acetic acid stress (Fig. 6H). The DEG analysis of the evolved Ent ev16 also showed upregulated expression of *CAR1* (2.24-fold) and *CAR2* (2.46-fold), which are involved in arginine catabolism and ornithine/urea biosynthesis (Fig. 6D and Table 4). The *CAR1* gene is involved in nitrogen catabolite repression (NCR), triggered by the GATA transcriptional activator Arg80, which controls *ARO9* and *ARO10* expression (ter Schure et al. 2000). However, the *car2*Δ strain showed impaired cell survival upon acetic acid stress when compared to the wild-type *S. cerevisiae* (BY4742) strain (Fig. 6H). A lack of arginine catabolic genes appears to slow growth under environmental stress, such as at high temperatures (Jarolim et al. 2013). Additional genes in arginine biosynthesis (*ARG1*, *ARG5,6*, and *CPA2*) showed downregulated expression (4.02-, 2.85-, and 2.02-fold, respectively) (Fig. 6D and Table 4). We also observed a downregulation of *ARG5* and *ARG6* expression, possibly resulting in the accumulation of *N*-acetyl-glutamate and glutamate, whereas *CPA2* downregulation may prevent the conversion of glutamine to carbamoyl-P, leading to the accumulation of glutamine inside the cells. In addition, *ARG1* inhibition may prevent arginine biosynthesis. The evolved Ent ev16 might have limited arginine biosynthesis and accumulate glutamate as a key precursor in amino acid biosynthesis. This may drive the TCA cycle and boost the generation of energy and cofactors to promote acetic acid resistance.

Strain Ent ev16 showed downregulated expression of *GDH1* (2.66-fold) and *GLN1* (5.94-fold), which are involved in the conversion of α-ketoglutarate to glutamate and further to glutamine, whereas the upregulated expression of *GDH3* (2.36-fold) facilitates the synthesis of glutamate (Fig. 6D and Table 4). In particular, only amino acids glutamate and aspartate promote acetic acid resistance through the pentose phosphate pathway by generating pentose phosphates and NADPH for the synthesis of nucleic acids, fatty acids, and glutathione (GSH) as well as the improved export of acetic acid in the bacterium *Acetobacter pasteurianus* (Yin et al. 2017). Furthermore, the evolved Ent ev16 strain displayed upregulated expression of the *BNA1* gene (2.14-fold), which is required for the de novo biosynthesis of nicotinamide adenine dinucleotide (NAD), an essential cofactor for cellular redox reactions and energy, suggesting that the evolved Ent ev16 strain adapted to acetic acid stress through the activation of redox reactions and energy metabolism (Fig. 6D and Table 4). In contrast, there was a downregulation of *BNA3*, which is involved in the conversion of kynurenine to kynurenic acid, an intermediate in NAD biosynthesis, as reported for *ARO9* in the kynurenine (de novo) pathway (Ohashi et al. 2013). Additionally, the downregulation of genes involved in leucine and valine biosynthesis, namely, *LEU1* (6.20-fold), *BAT1* (4.16-fold), *LEU2* (3.08-fold), *ILV3* (2.45-fold), and *ILV5* (2.06-fold), was found (Fig. 6D and Table 4). Downregulation of *ILV3* and *ILV5* in the evolved Ent ev16 strain reduced the conversion of pyruvate to 2-ketoisovalerate or 2-keto-3-methyl-valerate for valine, leucine, and isoleucine biosynthesis (Yuan et al. 2017). The expression of the aromatic amino acid transporter gene *TAT1* was upregulated (2.67-fold) (Fig. 6D and Table 4). This gene is involved in valine, leucine, isoleucine, and tyrosine amino acid transport (Bianchi et al. 2019). *tat1*Δ exhibited a low ability to survive under acetic acid stress, suggesting that *TAT1* is a key gene in the adaptive acetic acid tolerance of yeast (Fig. 6H).

The expression of genes involved in sulfur amino acid methionine and cysteine biosynthesis was downregulated in evolved Ent ev16. Additionally, we observed the downregulated expression of the homoserine biosynthesis gene *HOM3* (2.24-fold), which is involved in the conversion of aspartate to aspartate 4-PO_4_, a precursor in the biosynthesis of homoserine and other amino acids such as threonine, methionine, and glycine (Fig. 6D and Table 4). Significant changes in the expression of genes involved in the sulfate assimilation pathway (*SUL1*, *SUL2*), methionine biosynthesis (*MET32*, *MET28*, *MET3*, *MET14*, *MET16*, *MET5*, *MET10*, *MET17*, *MET6*, *SAM1*, *STR3*, *MTD1*), cysteine biosynthesis (*MET17* and *CYS3*), and glycine biosynthesis (*GCV1* and *GCV2*) were observed for Ent ev16 (Fig. 6D and Table 4). A spot test indicated that the *su11*Δ, *met17*Δ, and *gcv2*Δ strains showed reduced survival upon acetic acid stress (Fig. 6G, H), suggesting their importance in acetic acid tolerance. The upregulated expression of *AGX1* (2.64-fold), which controls glyoxylate and glycine conversion, and the downregulated expression of *SHM2* (27.48-fold), which is involved in glycine and serine conversion, indicated balanced glycine and serine levels in the cytoplasm (Fig. 6D and Table 4). Serine biosynthesis was also impeded via the downregulated expression of *SER3* (3.81-fold), *SER33* (2.75-fold), *SER1* (2.69-fold), and *SER2* (2.52-fold) (Fig. 6D and Table 4), blocking phosphoglycerate passage to the serine biosynthetic pathway.

Numerous “de novo” purine nucleotide biosynthesis genes were downregulated in the evolved Ent ev16 strain during acetic acid stress, such as *ADE4* (3.28-fold), *ADE5*,*7* (3.92-fold), *ADE6* (3.58-fold), *ADE2* (2.94-fold), *ADE1* (5.42-fold), *ADE13* (2.39-fold), *ADE17* (6.72-fold), *ADE3* (2.28-fold), *ADE12* (2.29-fold), and *HPT1* (3.45-fold), resulting in the suppression of both purine precursors adenosine and guanosine monophosphate (AMP and GMP) (Fig. 6D and Table 4). In Ent ev16, blockage of purine biosynthesis starts with the downregulation of *ADE4*, which encodes phosphoribosyl pyrophosphate amidotransferase. *ADE17* encodes enzymes of “de novo” purine biosynthesis; its expression was most significantly downregulated in the presence of acetic acid in the ancestral Ent, suggesting that *ADE17* suppresses the synthesis of inosine monophosphate (IMP) as the first step in purine nucleotide synthesis (Fig. 6E). *ADE17* expression was downregulated in the strain Ent ev16 (7.69-fold) compared with the ancestral Ent strain (Fig. 6F), but the deleted *ade17*Δ strain showed no growth defects in the presence of acetic acid (Fig. 6G).

The *IMD3* gene, which encodes IMP dehydrogenase, was downregulated (2.10-fold), preventing the conversion of IMP to xanthosine monophosphate (XMP) and, subsequently, to GMP (Fig. 6D and Table 4). In addition, the expression of *FCY2*, encoding a cytidine transmembrane transporter that mediates purine biosynthesis (adenine, guanine, and hypoxanthine), was downregulated (3.05-fold) (Fig. 6D and Table 4). Fcy2 may be required for the import of adenine, guanine, and hypoxanthine upon adenine defection (Guetsova et al. 1997). Phosphoribosyl pyrophosphate (PRPP) is not only a precursor in purine biosynthesis but also a substrate for histidine biosynthesis (Winkler and Ramos-Montañez 2009). The expression of *HIS1*, *HIS4*, and *HIS7* was downregulated (3.16-, 4.91-, and 2.91-fold, respectively) (Fig. 6D and Table 4). These *HIS* genes of histidine biosynthesis, particularly *HIS4*, play a role as multifunctional enzymes containing phosphoribosyl-ATP pyrophosphatase, which catalyze various steps in histidine biosynthesis (Keesey Jr et al. 1979). In the 9th and 10th steps of histidine biosynthesis, it is necessary to use NAD as a coenzyme. The evolved Ent ev16 did not use NAD in amino acid biosynthesis, resulting in the accumulation of NAD or in the use of other pathways. The DEG analysis of Ent ev16 showed the upregulated expression of *FMS1* (2.40-fold), which is involved in polyamine oxidase, especially in spermidine catabolization to β-alanine and pantothenic acid as a precursor of acetyl-CoA, which is related to lipid metabolism. This suggests that evolved yeast may develop tolerance by promoting the synthesis of key intermediates required for the construction of cell structures such as lipids. *TPO4* expression was also upregulated (2.26-fold); this gene is involved in spermidine and putrescine transport and secretion (Fig. 6D and Table 4). Recently, *TPO2* and *TPO3* expression has been reported to be relevant in acetate secretion, supporting acetic acid resistance (Zhang et al. 2022). The Ent ev16 strain expressed high levels of *TPO4*, which may improve acetic acid tolerance via acetate secretion.

Interestingly, the expression of some genes in thiamine biosynthesis (vitamin B1) was upregulated, including *THI13* (2.70-fold), *THI4* (5.07-fold), and *THI5* (2.42-fold) (Table 4 and Fig. 6D). The genes *THI13* and *THI5* are involved in the conversion of histidine and pyridoxal-5-P to the thiamine precursor hydroxymethylpyrimidine (HMP), resulting in the production of thiamine monophosphate (TMP) as a precursor for thiamine biosynthesis. The *THI4* gene is responsible to convert L-glycine, thiazole synthase, and NAD^+^ as substrates to 4-methyl-5-(β-hydroxyethyl) thiazole phosphate, which is an intermediate in thiamine biosynthesis (Perli et al. 2020). Upregulation of thiamine biosynthesis in the evolved Ent ev16 strain indicated cellular adaptation to acetic acid by increasing the production of thiamine as an essential cofactor enzyme in carbohydrate, amino acid, and lipid metabolism in probiotic yeasts. According to a previous report, thiamine promotes oxidative stress resistance in the yeast *Schizosaccharomyces pombe* (Kartal and Palabiyik 2019).

## Discussion

Laboratory evolution has been used as an engineering strategy for the selection of high-performing microbes with desirable traits, including tolerance to organic acids. Evolutionary adaptation to environmental stress is enabled by dynamic genetic and phenotypic changes observed in several organisms, including microbes (González-Ramos et al. 2016; Voordeckers et al. 2015). The medically important yeast *S. boulardii* is well known for its relative tolerance to acidic environments. Despite the 99% genomic sequence similarity to its close relative *S. cerevisiae* (Khatri et al. 2017), *S. boulardii* displays a unique probiotic property with the antipathogenic ability to produce antimicrobial compounds such as acetic acid (Moradi et al. 2018; Offei et al. 2019). Here, we identified some unique mutations of the evolved strains of *S. boulardii* using NGS (next-generation sequencing) and transcriptomic analyses associated with high acetic acid-induced stress tolerance at concentrations up to 7 g/L (Figs. 1 and 2). Notably, the evolved strains produced much higher levels of the stress-buffering agents glycerol and acetic acid in the culture media but produced low levels of ethanol (Fig. 4 and Table 1). When compared to its ancestral strain *S. cerevisiae*, which produced more ethanol as a key metabolite, our results suggest a metabolic shift in *S. boulardii* from ethanol fermentation to acid formation at this pH. The low ability of *S. cerevisiae* to generate biochemical compounds other than ethanol has been suggested to be linked to its metabolic flux (Yang et al. 2022). Since glycerol and acetic acid are also key precursors in fatty acid metabolism and plasma membrane components, their concentrations are vital for the maintenance of homeostasis and cell survival under stressful conditions.

In this study, four identified mutations involved in acetic acid stress adaptation are shared by the evolved strains Ent ev16 and ev17, which carry the acetic acid-tolerant phenotype. The four mutations include homozygous *oaf1* and *dan4* mutations as well as heterozygous *pan1* and *thi13* mutations (Table 3). First, the *pan1*^Thr486_Ser487insThrGlyMetMetProGlnThr^ and *pan1*^Gln1101_Pro1102insProThrGln^ mutations present disruptive in-frame insertions of amino acids (Fig. 5). Pan1 is involved in the regulation of actin cytoskeleton organization, which is essential for endocytosis regulation (Duncan et al. 2001). Stress adaptation is, in fact, closely associated with endocytosis, a process in which the plasma membrane invaginates to form vesicles to bring substances into cells (López-Hernández et al. 2020). Pan1 is also essential for the interactions between cells and the environment. Since the plasma membrane harbors elaborate transmembrane proteins, including transporters, channels, and receptors on the cell surface, they are important for maintaining ion homeostasis and initiating complex signaling cascades in response to adverse environmental stress (López-Hernández et al. 2020). In addition, Pan1 also regulates membrane turnover through the endocytic signaling pathway, which significantly affects the ubiquitin homeostasis of recycled or degraded proteins in the vacuole (Lee et al. 2017). Acetic acid stress can transiently activate Hog1 mitogen-activated protein kinase to phosphorylate aquaporin Fps1 to target this channel for endocytosis and degradation in the vacuole (Mollapour et al. 2008). A loss of Hog1 and Fps1 through a T231A and S537A double mutation generates a general loss of the endocytosis of cell surface proteins (Doa4 and End3), resulting in cell resistance to acetic acid stress (Mollapour et al. 2008).

Pan1 contains an EF-hand calcium-binding domain for the assembly of the Pan1-Sla1-End3 complex. Together, these proteins regulate the interaction between the actin cytoskeleton and endocytic compartments, which is required for membrane and cell wall morphogenesis (Tang et al. 2000; Tang and Cai 1996; Toshima et al. 2016). An altered function of the mutated Pan1 protein under acetic acid stress may lead to activation of endocytosis or membrane protein turnover of Fps1, thereby disrupting the Hog1 stress signaling pathway. The Pan1 protein is also phosphorylated and regulated by Hog1 in response to osmotic stress, suggesting its direct involvement in stress adaptation (Reiter et al. 2012). As shown by the qRT-PCR experiment, acetic acid induced the expression of these endocytic *PAN1*, *SLA1*, and *END3* genes by at least 2-fold in the evolved Ent ev16 strain compared to the noninduced condition (Fig. 5G). However, this induction is not observed in the ancestral strain Ent, suggesting a differential upregulation of *PAN1* in the acid-tolerant strain in *S. boulardii*. Due to the essentiality of the *PAN1* gene, the deletion of *PAN1* is lethal. Nevertheless, deletion of either the *SLA1* or the *END3* gene increased cell sensitivity to acetic acid (Fig. 5A), suggesting the involvement of the Pan1-Sla1-End3 complex in conferring acid tolerance.

Enhanced cellular adaptation to acidic environments resembling gastrointestinal conditions is associated with alterations in genes linked to cell surface systems on the plasma membrane and the modification of cell wall components and structures, as well as metabolism and transport (Ribeiro et al. 2022a). Some of the genes linked to these cellular adaptations have been found to carry mutations in the genome of evolved *S. boulardii* strains (Tables 3 and 4). As shown here, the *dan4*^Thr192del^ mutation allele presented a threonine deletion at position 192. This alteration might affect the function of mannoprotein and cell wall metabolism, as shown by the increased acetic acid sensitivity of the *dan4*Δ strain (Fig. 5A). When compared to the nonprobiotic relative *S. cerevisiae*, *S. boulardii* has a thicker cell wall, which might explain its exceptional probiotic properties and tolerance to various stress conditions (Hudson et al. 2016; Santovito et al. 2018). Although less is known regarding the role of *DAN4*, it is related to the Tir protein family of cell wall mannoproteins, whose expression is downregulated under acidic conditions. Indeed, RNA-seq identified changes in the expression of 20 genes associated with cell wall synthesis and organization, such as Chs3 chitin synthase and cell wall proteins (Table 4). Moreover, *end3*Δ is sensitive to acetic acid stress (Fig. 5A), suggesting functional loss of cell wall integrity. In the yeast *Candida albicans*, the mutant *end3* strain displays reduced clathrin-mediated endocytosis and subsequently impaired cell wall integrity, protein secretion, hyphal formation, and virulence-related processes (Rollenhagen et al. 2022). In *S. cerevisiae* strains, a lack of cell wall integrity and cell wall remodeling affect cell survival under acetic acid stress (Rego et al. 2014). Accordingly, *S. cerevisiae* strains display cell wall resistance under low pH and increased expression of *GAS1*, which is involved in (1→3)-β-D-glucan chain elongation under acetic acid induction (Ribeiro et al. 2021). The expression of ergosterol transporters affects yeast cell walls during adaptation to acetic acid (Ribeiro et al. 2022b). Thus, such physical and functional changes associated with cell wall remodeling and maintenance of *S. boulardii* are worth further investigation, as they are responsible for improved stress resistance and protection against external stimuli.

Next, the heterozygous mutation *thi13* ^Thr332Ala^ occurred in the *THI13* gene that belongs to the *THI5* family (*THI5*, *THI11*, *THI12*, and *THI13*) and is required for the biosynthesis of hydroxymethylpyrimidine (HMP), a precursor of thiamine (Wightman and Meacock 2003). Thiamine, or vitamin B1, is an essential nutrient indispensable for the normal growth and development of various organisms, including humans. In yeast, *THI13* expression is differentially expressed under low pH values, such as under furfural stress (Goud and Ulaganathan 2019). The involvement of thiamine in the maintenance of the redox balance in yeast cells under oxidative stress by shifting energy generation from fermentation to respiration has been reported elsewhere (Wolak et al. 2014). An isolated *S. cerevisiae* strain with increased acidity tolerance displays enhanced vitamin B1 and B6 biosynthesis, suggesting that thiamine biosynthesis or metabolism contributes to tolerance to low pH stress (Wu et al. 2022). Nevertheless, the rationale for acetic acid tolerance by mutation of the *THI13* gene, which is involved in thiamine metabolism, remains unclear, and the involvement of the *THI13* gene in antioxidation processes remains speculative.

To better understand the correlation, RNA-Seq was used to identify the rewiring of transcripts of genes involved in the oxidative stress response and amino acid metabolism. Regarding oxidative stress protection, the expression of genes encoding enzymes such as oxidoreductase, peroxidase, glutathione transferase, and reductase is activated. At the same time, mitochondrial aldehyde dehydrogenase, which is involved in acetate formation, is downregulated by acetic acid stress in the tolerant evolved strain compared to the ancestral strain. These findings indicate another important adaptive mechanism (Table 4). For amino acid metabolism, increased expression of many genes encoding an aromatic amino acid-requiring enzyme of the first step in the Ehrlich pathway (*ARO10*), thiazole synthase (*THI4*), and a protein for the synthesis of the thiamine precursor HMP (*THI13*), with a previously identified mutation, was found (Table 4). Decreased expression of several amino acid metabolic genes, such as those involved in the metabolism of serine, methionine, glycine, adenine, arginine, isoleucine, leucine, and histidine, as well as in generating precursors for de novo purine, pyrimidine, amino acid, and lipid biosynthesis, was identified (Table 4). Importantly, amino acid metabolism is closely linked to the tricarboxylic acid cycle (TCA), which is the main source of energy, metabolites, and cofactors during adaptation to stress. Additional changes in genes involved in the transport of ions, vitamins, and metabolites, including amino acids, fatty acids, sugars, and oligopeptides, were also observed (Table 4). The differential gene expression of many biological processes reflects the complex metabolic state of cells, as shown by the metabolomic changes that underpin the molecular nature of the stress response.

Finally, the *oaf1*^Ser57Pro^ mutation is found in the genome of the evolved Ent ev16 strain of *S. boulardii*. This alteration may help the evolved cells adapt to acetic acid stress. A mutated Oaf1 transcription factor could affect acid homeostasis and hence fatty acid metabolism, which contributes to the functional plasma membrane and membrane-bound proteins. Notably, the robustly evolved *S. boulardii* strains also secreted high levels of acetic acid during high glucose fermentation (Fig. 3). This is correlated with the observed increased glycerol accumulation and induced expression of the glycerol biosynthetic *GPP1* gene, which has been previously shown to protect yeast cells from acid stress (Lawrence et al. 2004). Because acetic acid is more frequently oxidized than ethanol, while glycerol is more reduced (Eglinton et al. 2002), therefore, the higher flux toward glycerol in the evolved yeast strains is perceived as less detrimental to yeast cells. Modulation of metabolic flux may be the adaptive mechanism to effectively maintain the intracellular redox balance and reduce the accumulation of toxic ethanol and acetic acid in probiotic yeasts. Similarly, overexpression of the *GPD1* gene has been observed in the study of wine yeasts with increased glycerol production. The authors report that a reduction in ethanol is accompanied by elevated concentrations of undesirable metabolites, including acetic acid (Remize et al. 1999). This is due to a perturbation in the redox balance of the high-glycerol/low-ethanol in the engineered wine yeast. Ald aldehyde dehydrogenase enzymes later help to restore the yeast’s redox balance by reducing coenzymes NAD^+^ or NADP^+^ during the oxidation of acetaldehyde to acetic acid and acetoin (Eglinton et al. 2002).

The high glycerol level is most likely a key factor that contributes to better tolerance to acetic acid stress in the evolved strains. The increased glycerol biosynthesis might involve the Hog1 regulator and the glycerol transporter Fps1, which is responsible for glycerol transport under environmental osmolarity or acidity stress (Babazadeh et al. 2014; Mollapour and Piper 2007). Since glycerol is a small, uncharged molecule that can easily penetrate lipid membranes, it is lost from cells via passive diffusion, following its concentration gradient (Duskova et al. 2015). The evolution of wine yeast strains for over 200 generations resulted in a reduction in the ethanol content of 1.3% (v/v) and a 41% (v/v) increase in glycerol yield under nonstress cultivation conditions (Tilloy et al. 2014). In addition to glycerol, acetic acid, acetaldehyde, and acetoin are also overproduced during osmotic stress (Kutyna et al. 2010). In previous studies, acetic acid stress triggered the activation of Hog1, which is involved in osmoregulation, leading to the accumulation of glycerol (Guaragnella et al. 2019; Nevoigt and Stahl 1997). The cellular accumulation of ergosterol and the increased level of the unsaturation index of fatty acids in the plasma membrane also help improve yeast tolerance to organic acids (Guo et al. 2018).

Furthermore, glycerol is secreted into the culture media at a high level, in parallel with the increased expression levels of the *GPP1* gene for glycerol biosynthesis as well as the *OLE1* gene for fatty acid synthesis in the acetic acid-resistant evolved Ent ev16 *S. boulardii* strain (Fig. 5G). This suggests metabolic rewiring and regulation in favor of the modification of the cellular membrane composition and architecture. The *OLE1* gene is the target of the Oaf1 regulator that controls lipid and fatty acid metabolism (Bergenholm et al. 2018) as well as nonfermentable substrates (Turcotte et al. 2010). An *OLE1*-overexpressing strain with increased levels of oleic acids displayed an increase in the oleic acid (18:1n-9) content in the membrane and better resistance to acetic, formic, and levulinic acids (Guo et al. 2018).

Next, CRISPR/Cas technology will be necessary to construct the pan1* mutation or other identified SNPs to further prove the involvement of these mutations in acetic acid resistance. Afterward, the identified mutations could be applied to strain engineering for improved acetic acid tolerance as well as acid or glycerol production in other yeast strains, including commercial strains. Indeed, weak acids have wide ranges of applications in pharmaceutical and biochemical industries as food preservatives (e.g., acetic, propionic, benzoic, and sorbic acids), herbicides, and drug precursors for anticancer, antimalaria, and immunosuppressor drugs. Understanding the mechanisms of toxicity and tolerance to weak acid stress in probiotic yeast may help identify new drug targets and extend applications of yeast strains in health care as well as food and beverages. Supplements with probiotic *S. boulardii* could be a powerful therapeutic option for the treatment or alleviation of life-threatening diseases such as cirrhosis and ethanol-associated liver cancer (Chen et al. 2022).

## Supplementary information

ESM 1 (PDF 742 kb)

## Data Availability

Full details are given in the Supplementary Materials, Tables S1–S5, and Figs. S1 and S2. The genome sequencing data and the RNA-seq data are deposited in the NCBI database, as mentioned in the “Materials and methods” section. Additional data will be made available upon reasonable request.
