# Supplementary material for: New biomarkers underlying acetic acid tolerance in the probiotic yeast Saccharomyces cerevisiae var. boulardii
Source: Appl Microbiol Biotechnol. 2024 Jan 19;108:153. doi: 10.1007/s00253-023-12946-x (PMC10799125; doi:10.1007/s00253-023-12946-x)
Supplement: Supplementary file 1 [file 253_2023_12946_MOESM1_ESM.pdf]

Supplemental Material

Applied Microbiology and Biotechnology

New biomarkers underlying acetic acid tolerance in the probiotic yeast *Saccharomyces cerevisiae* var. *boulardii*

Wiwan Samakkarn[1], Paul Vandecruys[2,3], Maria Remedios Foulquié Moreno[2,3], Johan Thevelein[2,3,4], Khanok Ratanakhanokchai[1,5], and Nitnipa Soontorngun[1]*

[1]Gene Technology Laboratory, Division of Biochemical Technology, School of Bioresources and Technology, King Mongkut's University of Technology Thonburi, Bangkhuntian- Chai Thale Rd., Tha Kham,Bangkhuntian,Bangkok 10150, Thailand.

[2]Laboratory of Molecular Cell Biology, Institute of Botany and Microbiology, KU Leuven, Leuven-Heverlee, Belgium.

[3]Center for Microbiology, VIB, Kasteelpark Arenberg 31, B-3001, Leuven-Heverlee, Flanders, Belgium.

[4]NovelYeast Bv, Open Bio-Incubator, Erasmus High School, Laarbeeklaan 121, B-1090, Brussels (Jette), Belgium.

[5]Pilot Plant Development and Training Institute, King Mongkut's University of Technology Thonburi, Bangkhuntian-Chai Thale Rd.,Tha Kham,Bangkhuntian,Bangkok 10150, Thailand.

* E-mail: nitnipa.soo@kmutt.ac.th, Telephone number: (+66)2-470-7753, Fax number: (+66)2-452-3479

Table S1 Primers for RT-qPCR analysis used in this study

Purpose	Gene	Primer sequence(s) (5’ to 3’)
Endocytosis	*PAN1*	AGCAAGCACAGTCCTTGC and CACCGGAAACAGTATTCGAACC
	SLA1	ACCAAGCTCCAGATGAGGA and CCTCGATTTCGGTAACGTTCC
	END3	GCAGACTTGCCTCCAAAGAA and CTCCGCCAGAGAATTTGGT
Signaling/MAPK	*TOR1*	GCAGCCTCATCTGGTTACG and GAGACATGCCCTGCATGAG
	SMI1	ATCACTCACGCAGAGGAAGA and GTGCATATACCGGTTGCACT
Fatty acid biosynthesis	*OLE1*	CATCTCCGAACAACCATGGA and ATCTCAATGGCCAGTGAGC
Glycerol biosynthesis	*GPD1*	GTGGGTGTTCGAAGAAGAG and ACAGGAGATAGCTCTGACG
	GPP1	GAAGTTCCAGGTGCTGTC and CGTTGTATTCACCGACTC
Transporter activity	*VBA5*	GGCTCGTTACAGGCTACAG and TCACCAATGGACGTGACC
	FRE7	GTCTGTTCGTGCAGGCATAA and
	YPT53	GTCTTCTAGCAATAGGCAGCG
	OPT2	AACCTACTATTGGTGCCGC and AGTCTTAGCGCTTGCTTCG
	ADE17	CGGAAGACGTTGCGTATATCC and
		CGGAATGTCTCCACTGGAAT
		GTGTGACTATTCCAGAAGCCG and
		TGTGGATTGGCACCATAACG

Table S2 Mapped data stats of *S. boulardii*

Strain	Ref. length	Mapped sites (≥ 1x)	Total reads	Mapped reads	Mapped bases	Mean depth
**Enterol (ancestral)**	12,001,065	11,966,515 (99.71 %)	6,326,040	5,383,980 (85.11%)	751,552,975	62.62
**Enterol ev16**	12,001,065	11,966,699 (99.71 %)	6,691,164	6,223,777 (93.01%)	877,736,951	73.14
**Enterol ev17**	12,001,065	11,968,794 (99.73 %)	6,927,114	5,913,452 (85.37%)	831,147,204	69.26

Table S3 Raw data stats of *S. boulardii* bases sequenced

Strain	Total read base (bp)	Total reads	GC (%)	Q20 (%)	Q30 (%)
**Enterol (ancestral)**	1,206,462,216	7,989,816	37.93	96.63	92.70
**Enterol ev16**	1,097,931,570	7,271,070	36.65	96.75	92.82
**Enterol ev17**	1,168,264,350	7,736,850	37.63	96.65	92.57

Table S4 Filtered data stats of *S. boulardii* bases sequenced

Strain	Total read base (bp)	Total reads	GC (%)	Q20 (%)	Q30 (%)
**Enterol (ancestral)**	1,007,457,971	6,927,114	37.55	99.11	97.07
**Enterol ev16**	918,284,036	6,326,040	36.27	99.09	97.00
**Enterol ev17**	970,684,577	6,691,164	37.34	99.07	96.95

Table S5 Summary of variant count of *S. boulardii*

Strain	Number of SNPs	Number of insertions	Number of deletions
**Enterol (ancestral)**	1,942	565	122
**Enterol ev16**	1,898	573	130
**Enterol ev17**	1,965	576	125

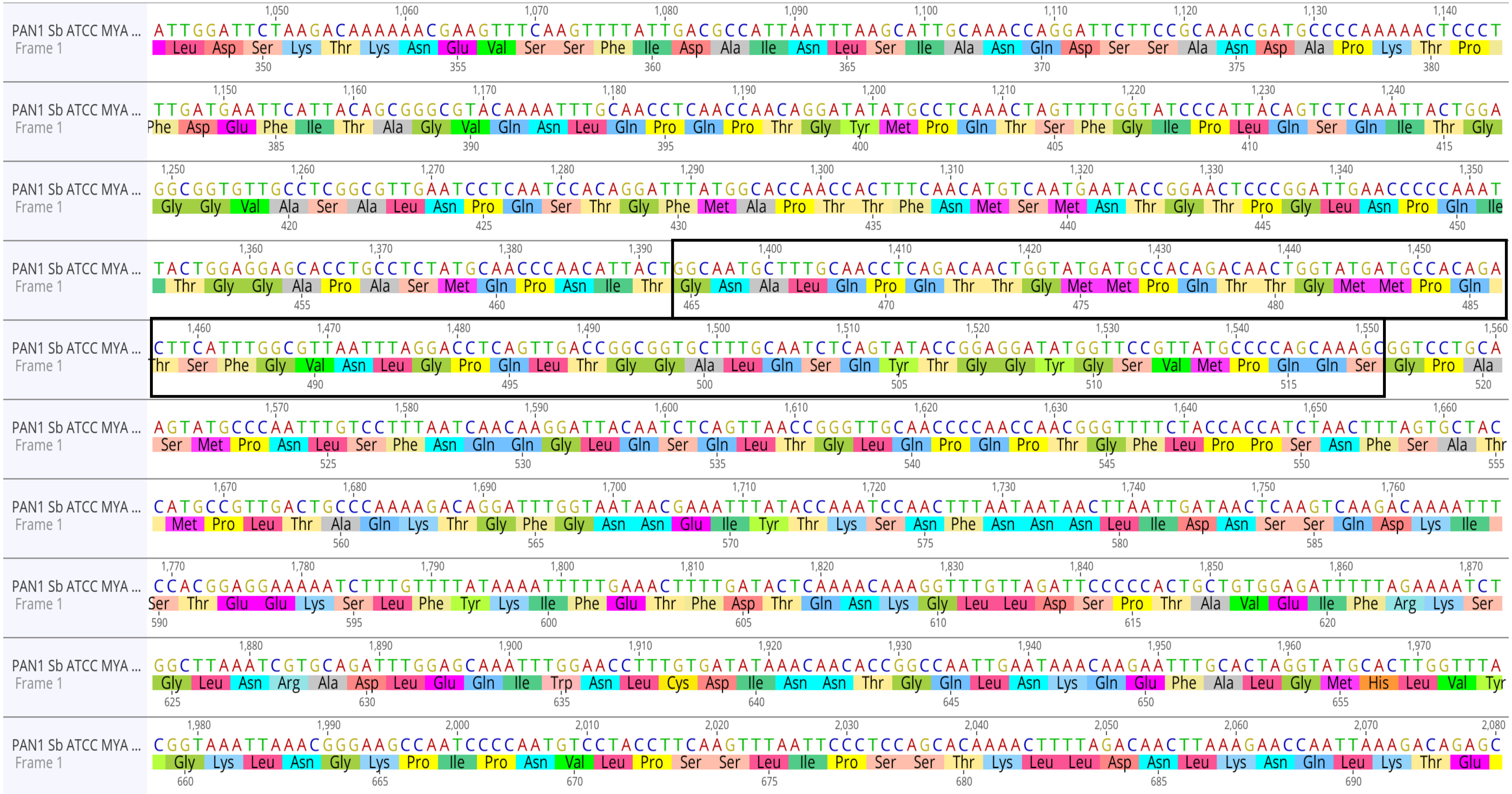

Fig. S1 The nucleotide and amino acid sequences of the *PAN1* gene in the yeast *S. boulardii* ATCC MYA-796. The ranges of sequence starting from nucleotide position 1,393 (amino acid at 465) were used to analyze the polymorphism in various evolved Enterol strains (black box) via polymerase chain reaction (PCR) amplification technique with specific oligonucleotides, which cover the mutation site.

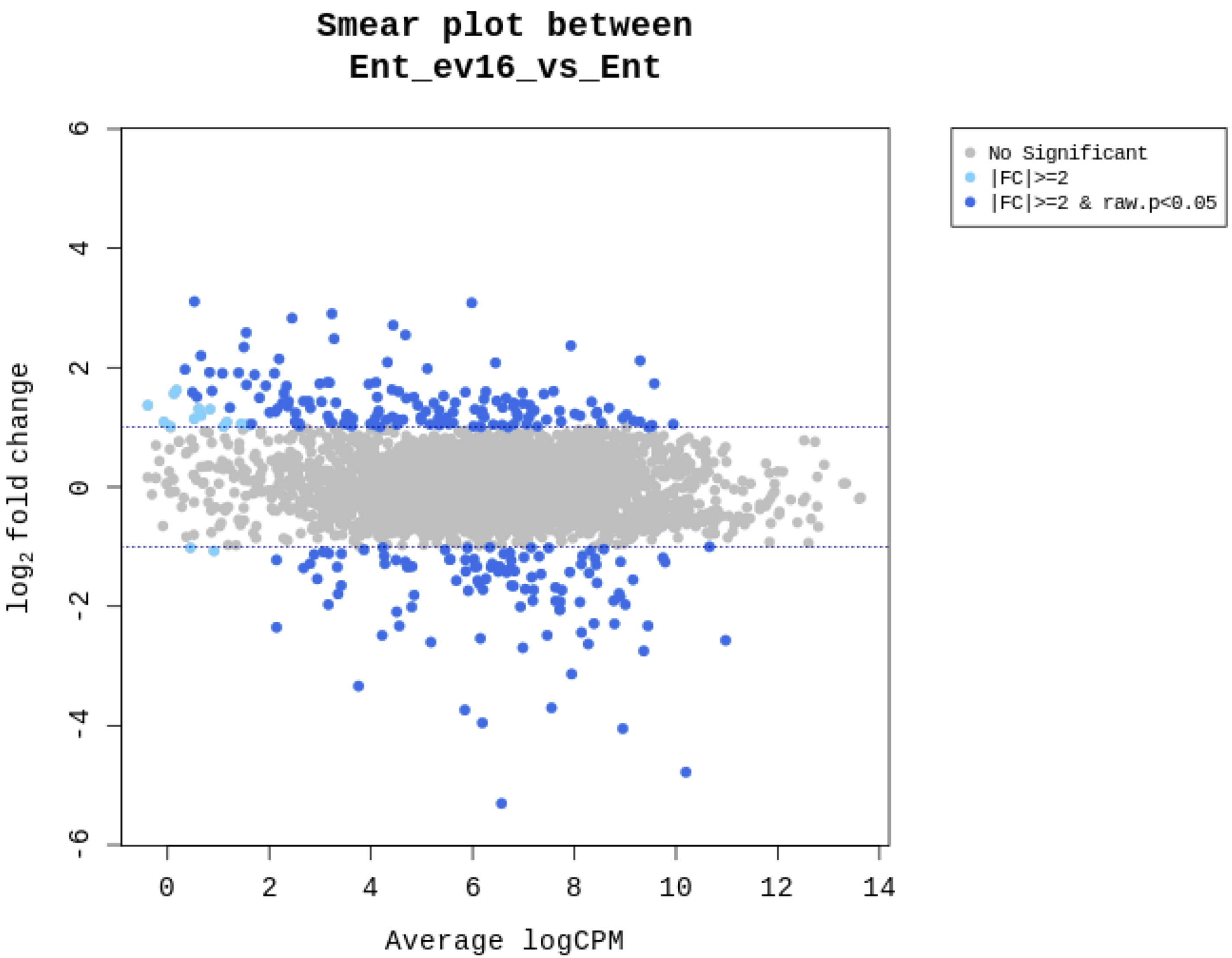

Fig. S2 The transcripts of DEGs in Enterol ev16 versus ancestral Enterol according to the overall average expression level (Smear); a smear plot is drawn. (X-axis: Average logCPM, Y-axis: log2-fold change)
